# Polar Localization of PhoN2, a Periplasmic Virulence-Associated Factor of *Shigella flexneri*, Is Required for Proper IcsA Exposition at the Old Bacterial Pole

**DOI:** 10.1371/journal.pone.0090230

**Published:** 2014-02-27

**Authors:** Daniela Scribano, Andrea Petrucca, Monica Pompili, Cecilia Ambrosi, Elena Bruni, Carlo Zagaglia, Gianni Prosseda, Lucia Nencioni, Mariassunta Casalino, Fabio Polticelli, Mauro Nicoletti

**Affiliations:** 1 Dipartimento di Scienze Sperimentali e Cliniche, Università “G. D'Annunzio”, Chieti, Italy; 2 Dipartimento di Sanità Pubblica e Malattie Infettive Università “Sapienza” di Roma, Rome, Italy; 3 Dipartimento di Biologia e Biotecnologie “C. Darwin”, Università Sapienza di Roma, Rome, Italy; 4 Dipartimento di Scienze, Università di “Roma Tre”, Rome, Italy; 5 Istituto Nazionale di Fisica Nucleare, Sezione di “Roma Tre”, Rome, Italy; University of Padova, Medical School, Italy

## Abstract

Proper protein localization is critical for bacterial virulence. PhoN2 is a virulence-associated ATP-diphosphohydrolase (apyrase) involved in IcsA-mediated actin-based motility of *S. flexneri*. Herein, by analyzing a Δ*phoN2* mutant of the *S. flexneri* strain M90T and by generating *phoN2*::HA fusions, we show that PhoN2, is a periplasmic protein that strictly localizes at the bacterial poles, with a strong preference for the old pole, the pole where IcsA is exposed, and that it is required for proper IcsA exposition. PhoN2-HA was found to be polarly localized both when *phoN2*::HA was ectopically expressed in a *Escherichia coli* K-12 strain and in a *S. flexneri* virulence plasmid-cured mutant, indicating a conserved mechanism of PhoN2 polar delivery across species and that neither IcsA nor the expression of other virulence-plasmid encoded genes are involved in this process. To assess whether PhoN2 and IcsA may interact, two-hybrid and cross-linking experiments were performed. While no evidence was found of a PhoN2-IcsA interaction, unexpectedly the outer membrane protein A (OmpA) was shown to bind PhoN2-HA through its periplasmic-exposed C-terminal domain. Therefore, to identify PhoN2 domains involved in its periplasmic polar delivery as well as in the interaction with OmpA, a deletion and a set of specific amino acid substitutions were generated. Analysis of these mutants indicated that neither the ^183^PAPAP^187^ motif of OmpA, nor the N-terminal polyproline ^43^PPPP^46^ motif and the Y155 residue of PhoN2 are involved in this interaction while P45, P46 and Y155 residues were found to be critical for the correct folding and stability of the protein. The relative rapid degradation of these amino acid-substituted recombinant proteins was found to be due to unknown *S. flexneri*-specific protease(s). A model depicting how the PhoN2-OmpA interaction may contribute to proper polar IcsA exposition in *S. flexneri* is presented.

## Introduction

Bacteria maintain a subcellular spatial organization that is specifically related to function. Spatial positioning of proteins has been shown to be critical to several bacterial cellular processes and bacteria have evolved different mechanisms in order to target proteins to specific location within the cell [1]. Several bacterial proteins essential to virulence of pathogens are known to localize to one or both poles. Type V secretion systems are an extensive family of large monomeric autotransporter outer membrane (OM) proteins, typically virulence factors, produced by Gram-negative bacteria [2], [3], [4]. Recent evidence indicates that autotransporters prevalently localized at the old pole of the bacterium where translocation across the OM appears to occur via specific conserved pathways also localized at the old pole of the rod [3], [5], [6].

*Shigella flexneri* causes bacillary dysentery in humans due to bacterial invasion and colonization of the colonic epithelium [7], [8]. The ability of *S. flexneri* to move within the eukaryotic cell cytoplasm and to spread infection inter-cellularly is due to the expression and exposition at the old bacterial pole of IcsA, a 120-kDa autotransporter protein encoded on the 220-kb virulence plasmid (pINV) [9], [10], [11]. Once IcsA is translocated across the OM, the exposed N-terminal α-domain interacts with the host actin regulatory proteins vinculin and neural Wiskott-Aldrich syndrome protein (N-WASP). N-WASP then recruits the host Arp2/3 complex to initiate polymerization of host globular actin into filamentous actin (F-actin) [12], [13], [14], [15], [16]. The assembly of F-actin in comet tails at the old pole of the bacterium initiates bacterial actin-based motility (ABM) [9], [13], [15].

Apyrase (PhoN2), is a *S. flexneri* ATP-diphosphohydrolase virulence-associated protein which belongs to the family of the non-specific bacterial acid phosphatases of class A (A-NSAPs) [17]. PhoN2 is encoded by *phoN2* (*apy*), a gene located on a highly conserved region (the *ospB-phoN2* operon) within the pINV of *Shigella* species and related enteroinvasive *Escherichia coli* (EIEC) strains [18], [19]. *ospB* and *phoN2* are co-transcribed as a 2 kb bicistronic, temperature-regulated mRNA from an upstream promoter that precedes *ospB* [19]. OspB is a type III secretion (TTS) system-secreted effector and transcription of the *ospB-phoN2* operon is regulated by the VirF/VirB cascade and by MxiE, in concert with IpgC [10], [20], [21]. Thus, even if PhoN2 is not a secreted effector, *phoN2* transcription is also up-regulated by MxiE when the TTS system is activated, suggesting that PhoN2 may be relevant in post-invasion events [21]. We have previously shown that PhoN2 is required, in a deoxynucleotide triphosphate-hydrolyzing activity-independent manner, for efficient inter-cellular spreading since a non-polar Δ*phoN2* mutant of the wild-type *S. flexneri* 5a strain M90T presented altered ABM and a small plaque phenotype [21]. The mechanism by which PhoN2 influences ABM and plaque size has not been elucidated yet. However, since PhoN2 possesses an exposed N-terminal polyproline (^43^PPPP^46^) sequence and proline-rich motifs have been reported to be involved in inter- and intra-molecular interactions, in protein folding and essential for virulence of various intracellular pathogens [22], [23], [24], [25], [26], we hypothesized that the ^43^PPPP^46^ motif of PhoN2 could directly interact with IcsA or indirectly with unknown accessory proteins necessary in assisting proper polar IcsA exposition [21].

To unravel this point we first analyzed the spatial localization of PhoN2 in *S. flexneri* and found that PhoN2 is a periplasmic protein even if this cellular compartment is known to be devoid of ATP and GTP molecules [2], the natural substrates of its catalytic activity. Thereafter, we demonstrated that PhoN2 localizes at the bacterial poles, mainly at the old bacterial pole, the pole where IcsA is exposed. Remarkably, we found that PhoN2 is required for proper IcsA exposition on the bacterial surface. Moreover, two-hybrid and cross-linking experiments allowed us to identify the periplasmic-exposed C-terminal domain of the OM protein OmpA as a strong PhoN2 interactor. Since PhoN2 polar localization was found to be independent from both IcsA and OmpA, a detailed mutagenesis analysis on key features of the PhoN2 amino acid sequence was performed. The results obtained demonstrated that the N-terminal polyproline ^43^PPPP^46^ motif along with the Y155 residue are critical for the stability of PhoN2. A model depicting how the PhoN2-OmpA interaction might contribute to the correct polar exposition of IcsA in *S. flexneri* is presented.

## Materials and Methods

### Bacterial strains, plasmid and growth conditions

Bacterial strains and plasmid used in this study are listed in Table S1. *S. flexneri* strains were plated on Trypticase soy broth agar (TSA) plates (BBL Microbiology Systems) containing 0.01% Congo red (Sigma). *E. coli* K-12 and *S. flexneri* strains were grown on Luria-Bertani (LB) broth [27] and Trypticase soy broth. Antibiotics (Sigma), where appropriate, were added at the following concentrations: 100 μg ml^−1^ ampicillin (Ap); 30 μg ml^−1^ chloramphenicol (Cm); 30 μg ml^−1^ kanamycin (Km); 100 μg ml^−1^ streptomycin (Sm); 5 μg ml^−1^ tetracycline (Tc). Unless otherwise stated, L-arabinose was added at a final concentration of 0.016% (wt/vol).

### DNA manipulations

DNA extraction, isolation of plasmids, restriction digestion, electrophoresis, purification of DNA fragments, construction of recombinant plasmids, transformation, transduction and immunoblotting were performed by standard methods [28]. The primers used for PCR amplifications and mutant constructions are listed in Table S2. Oligonucleotide primers were designed based on the available *S. flexneri* pINV and genome sequences (accession numbers AL391753 and CM001474, respectively). Thermal cycling conditions were as previously described [19], [21]. DNA sequence data were compared to known nucleotide and protein sequences using the BLAST Server (National Centre for Biotechnology Information, Bethesda, Md).

### Construction of mutant strains and recombinant plasmids

HND115 is a Δ*phoN2* non-polar deletion derivative of the *S. flexneri* serotype 5a strain M90T (Table S1). C-terminal HA-tagging of *phoN2* was achieved, as previously described [29]. Briefly, two DNA modules that begin with the HA-encoding sequences followed by a Km^r^ cassette flanked by FRT sites were amplified, using plasmid pSU315 as template, with primer pair PhoN2fw/PhoN2rv (Table S2). The *phoN2*::HA fusion was moved into wild-type M90T by P1 transduction, selecting for Km^r^ resistance. The Km^r^ cassette was eliminated, thus generating strain HNDHA10 *phoN2*::HA (Table S1), and the correct structure of the *phoN2*::HA fusion was verified by PCR and DNA sequencing. Full length *phoN2*::HA fusion was further PCR-amplified using the primer pair PhoN2HAfw/PhoN2HArv (Table S2), and cloned into the polylinker site of pBAD28, under the control of the P_BAD_ L-arabinose inducible promoter [30], generating plasmid pHND10 (Table S1). Full length *ompA*, together with its natural promoter, was PCR-amplified using total DNA preparations of the wild-type *S. flexneri* strain M90T as template and the primer pair DM1fw/DM2rv (Table S2). The PCR product ends were digested with *Kpn*I and *Hind*III and cloned into pBAD28 thus generating plasmid pOmpA.

An in frame deletion encompassing a DNA region encoding the ^43^PPPP^46^ motif of the *phoN2* gene (nucleotides 79–223) was generated by PCR using pHND10 DNA as template and the primer pair DS4fw/LPrv (Table S2). The generated PCR fragment was *Xho*I-digested, self-ligated and transformed into *E. coli* DH10b competent cells thus generating recombinant plasmid pHND11_Δ79–223_. DNA sequencing was performed in order to verify the introduction of the deletion within the *phoN2*::HA gene.

The QuickChange site-directed mutagenesis kit (Stratagene) was used to introduce amino acid substitutions within PhoN2-HA and OmpA using plasmids pHND10 and pOmpA DNA as templates. Primer pairs used to introduce the desired nucleotide substitutions are reported in Table S2. Following PCR, reaction mixtures were incubated with 10U of *Dpn*I (Invitrogen) and used to transform XL1-Blue competent *E. coli* cells. Transformants were selected by plating on LB plates supplemented with Cm and Ap for pHND10 derivatives, and with Tc for the OmpA derivative, thus generating recombinant plasmids pHND19_R192P_, pHND23_SPPP_, pHND14_PSPP_, pHND15_PPSP_, pHND16_PPPS_, pHND21_Y155A_ and pAAAOmpA (Table S1). All generated plasmids were verified by PCR and sequencing to assess the introduction of the desired nucleotide substitutions before being introduced into *S. flexneri* and *E. coli* K-12 strains by electroporation. The periplasmic localization of PhoN2-HA encoded by all pHND10-derivative recombinant plasmids was verified by subcellular fractionation and Western blot analysis.

A deletion encompassing the *ompA* gene was moved from the *E. coli* K-12 strain JW0940 [Keio collection; NBRP (NIG, Japan): *E. coli*] (Table S1) into the *S. flexneri* strains M90T and HND115 by P1 transduction, thus generating Δ*ompA* derivative strains HND92 and HND93, respectively (Table 1).

**Table 1:** Preys selected by the PhoN2 bait.

Prey	Start^a^	End^a^	Number of clones^b^	Common region^c^
OmpA (348 aa)	189	276	5	189–273
	185	304	2	
	43	275	2	
	188	299	1	
	51	273	1	
	72	277	1	
	186	284	1	
Adh (336 aa)	129	218	1	
AspRS (590 aa)	358	472	1	
EF-2 (665 aa)	114	264	1	

a Amino acid coordinates, with respect to the sequence of native proteins, of the first and last residue encoded by the insert in each prey plasmid.

b Number of independent clones containing identical prey fragments.

c Coordinates of the common region carried by all preys corresponding to the same protein.

### Real-time quantitative PCR

Total RNA was extracted as described by [31]. cDNA synthesis was performed using the Quantiscripts Reverse Transcriptase Kit (Qiagen) in 20 µl reaction mixtures, according to manufacturer's instruction. Mixtures contained 1 µg of DNase-treated total RNA preparations from wild-type *S. flexneri* M90T or from its Δ*phoN2* derivative strain HND115 complemented with plasmid pHND10. Real-time quantitative PCR was performed as previously described [32]. The following pairs of oligonucleotides were used: naF/naR for the *nusA* transcript; ApyF/ApyR for *phoN2* and *phoN2*::HA (Table S2).

### Preparation of protein extracts and cell fractionation

Equal numbers of exponentially-growing bacteria were harvested by centrifugation at 3,000×g for 20 min at 4°C. After washing, bacteria were suspended in 3 ml of 30 mM Tris-HCl (pH 8.0), 4 mM EDTA, 1 mM PMSF, 20% sucrose and 0.5 mg/ml lysozyme and incubated 2 min at 30°C. MgCl_2_ (10 mM final) was added to the bacterial solution and incubation was continued for 1 h at 30°C. At the end of the incubation period bacterial suspensions were centrifuged at 11,000×g for 10 min at 4°C and supernatants were stored (periplamic fractions). Pellets were suspended in 1 ml of ice-cold 30 mM Tris-HCl (pH 8.0), 10 mM MgCl_2_ and sonicated for 10 sec 8 times at 30 sec intervals on ice using the Soniprep MSE 150 apparatus (Sanyo). Cleared lysates were collected by centrifuging at 30.000×g for 30 min at 4°C. Supernatants were stored (cytosolic fractions) and pellets (membrane fractions) were solubilised in 1x Laemmli buffer [33]. Cytosolic and periplasmic fractions were concentrated by TCA-precipitation. Precipitated proteins were washed in 90% of acetone, suspended in 1x Laemmli buffer and stored at −30°C until use.

### PhoN2 stability assay: half-life determination

For *in vivo* assessment of PhoN2-HA stability, bacteria were grown to an A_600_ of 0.8 in LB, a sample was removed (time zero), and spectinomycin was added (final concentration 100 μg ml^−1^) to the remainder to stop protein synthesis. Equivalent samples were then removed 30, 60, 120 and 240 min later and whole cell extracts were subjected to Western blot analysis using a monoclonal anti-HA antibody (Sigma). Half-live was calculated by the standard equation for a first-order decay process.

### Apyrase activity assay

Apyrase activity of bacterial cleared lysates were assayed at 37°C in 200 μl of 0.1 M Tris-HCl (pH 7.5), 1 mM ATP, and 10 mM EDTA. The reaction was terminated by the addition of 1/10 volume of trichloroacetic acid and the Pi released was estimated as previously described [34], [35]. One unit of the enzyme corresponds to the release of 1 μmol of phosphate min^−1^ mg^−1^ of protein. Protein concentrations were determined by the method of Lowry, using a commercial kit (Bio-Rad).

### Tissue culture conditions and virulence assays

Culture and infection of HeLa cells were performed as previously described [21]. *S. flexneri* invasion of semi-confluent monolayers was carried out using the gentamicin protection assay, as previously described [19], [21].

### Immunofluorescent labelling

Exponentially-growing bacteria were washed and fixed to polylysine-treated cover-slips or used to infect HeLa cell monolayers. Bacteria on polylysine-treated cover-slips or inside infected HeLa cells were fixed for 15 min with a solution of 3.7% paraformaldehyde. IcsA exposition on the bacterial surface was determined by indirect immunofluorescence on fixed intact bacterial cells. All centrifugations were carried out at 5,000×g for 5 min followed by washes with 1 ml volumes. Briefly, 1 ml of fixed cells were centrifuged and washed three times with phosphate buffered saline (PBS). Bacteria were then blocked for 1 h at 37°C in 100 µl of foetal bovine serum (FBS). Rabbit polyclonal antibodies to IcsA (kindly provided by A. Phalipon) were added to a final dilution of 1∶100 in PBS supplemented with 10% normal goat serum and bacteria were incubated for 1 h at 37°C. At the end of the incubation period, bacteria were washed three times in PBS, suspended in 100 µl of PBS containing a 1∶200 dilution of TRITC-labelled goat anti-rabbit IgG, and incubated for 30 min at room temperature. After incubation cells were washed three times with PBS and aliquots centrifuged onto polylysine-treated cover-slips at 2,000 rpm for 10 min.

PhoN2-HA and PhoN2-HA recombinant derivative proteins were immune-detected using anti-HA monoclonal antibody (clone 12AC5). Briefly, fixed bacteria were first permeabilized for 10 min at room temperature with a 0.25% solution of Triton X-100, in PBS, then for 5 min with 0.3 mg ml^−1^ of lysozyme in TE buffer, and then blocked for 1 h a 37°C with 1% normal goat serum. Triton X-100 permeabilization, followed by lysozyme treatment, was also used to immunodetect PhoN2-HA in bacteria within infected HeLa cells. Indirect immunofluorescence was performed by incubating bacteria for 30 min at room-temperature in the presence of TRITC-labelled goat anti-rabbit IgG (IcsA) and with FITC-labelled goat anti-mouse IgG (PhoN2) (Jackson ImmunoResearch).

Double indirect immune-fluorescence labelling of both IcsA and PhoN2-HA was carried out as follows: after IcsA labelling, washed bacteria were permeabilized 10 min with 0.25% Triton X-100 in PBS and 5 min with 0.3 mg ml^−1^ of lysozyme, in TE buffer, and incubated 1 h at room-temperature in the presence of anti-HA antibody. Single images were recorded with a Leica DM5000B microscope equipped with the Digital FireWire Color and Black&White Camera Systems Leica DFC300FX and Leica DFC340FX, respectively, and processed using the Leica Application Suite 2.7.0.R1 software (Leica).

The percentage of bacteria labeled in each experiment (labeled/total numbers of bacteria) were determined by counting in the same microscopic field under immunofluorescence and phase-contrast visualization. For each experiment, at least 200 bacteria were counted. Counting was performed blinded to the particular preparation. The data presented were always from three independent experiments.

### Yeast two-hybrid analysis

Prokaryotic proteins interacting with PhoN2 were identified using the two-hybrid technique in yeast (Matchmaker Library Construction and Screening kit; Clontech). The *phoN2* gene was PCR amplified with the primer pair HTYPHfw and HTYPHrv (Table S2), double digested with *Nco*I/*Bam*HI, and cloned in-frame into the polylinker site of the bait plasmid pGBKT7 digested with the same enzymes, to create plasmid pGBKT7/*phoN2* (Table S1). pGBKT7/*phoN2* was subjected to sequence analysis and immunoblotting to verify PhoN2 expression (data not shown). cDNA library of wild-type *S. flexneri* strain M90T (Table S1) was generated by the SMART technology (Clontech), following manufacturer's instructions. Briefly, 2.0 ml of exponentially-growing bacteria, grown in LB broth, were harvested by centrifugation and total RNA was extracted by using the RiboPure-Bacteria kit (Ambion). Double-strand (ds) DNA was obtained by reverse transcription of 2 µg aliquot of a total RNA preparation of strain M90T using the CDSIII/Random primer, the SMART III primer, and Moloney Murine Leukemia Virus (MMLV) reverse trascriptase. The library was amplified by Long-Distance PCR using the Advantage 2 PCR Kit and 5′- and 3′-PCR primers (Clontech). The cDNA library, the *Sma*I linearized pGADT7-Rec and pGBKT7/*phoN2* plasmids were used to transform simultaneously *Saccharomyces cerevisiae* strain AH109 made competent by treatment with lithium acetate, as recommended by the manufacturer (Clontech). The interaction between the bait plasmid pGBKT7/*phoN2* and the prey-generated pGADT7-Rec-based recombinant plasmids containing the cDNA library was screened by plating on SD minimal medium lacking leucine, tryptophan, histidine and adenine. Colonies arising after 5 days of incubation at 30°C, were streaked twice on the same selective plates and subjected to α-galactosidase quantitative assay to measure the expression of the *MEL1* gene (Yeast Protocols Handbook, Clontech). Plasmid DNA preparations were obtained from well isolated α-galactosidase-positive colonies grown on liquid SD medium lacking leucine, tryptophan and histidine. Plasmid preparations of independent clones were used to transform competent *E. coli* strain DH10b. Independent prey plasmids (pGADT7-Rec carrying cDNA inserts) were selected on LB agar plates supplemented with 30 µg ml^−1^ of Km. Plasmid DNA preparations of *E. coli* Km^r^ transformants were used to sequence the cDNA inserts. *S. flexneri* genes were identified by BLAST analysis.

### *In vivo* cross-linking experiments

Bacteria were grown at 37°C in LB, in the presence or not of L-arabinose, to an OD_600_ of 0.8 and harvested by centrifugation (13,000×g, 2 min). Bacterial pellets were washed twice with an equal volume of ice-cold PBS pH 6.8, and finally resuspended to an OD_600_ of 0.5. Cross-linking was achieved by adding formaldehyde to a final concentration of 1%. Aliquots of 1 ml were incubated for 0 and 15 min at room temperature, followed by glycine quenching (0.125 mM). Cross-linked samples were centrifuged at 13,000×*g* for 2 min, and the pellets were resuspended in 50 µl of 2x Laemmli buffer [33] and heated either at 37°C for 10 min or at 95°C for 20 min to maintain or to break the chemical cross-links, respectively.

### SDS-PAGE and immunoblot analysis

Equal amounts of proteins were separated on 12.5% SDS-PAGE, transferred to PVDF membranes (Hybond-P, Millipore) and analyzed by immunoblotting. A protein molecular weight marker (Pierce) was included in each electrophoresis run in order to determine the molecular weight of the proteins. Immunoblotting was carried out with rabbit polyclonal anti-OmpA (gift from Dr. N. Prasadarao) and monoclonal anti-HA (Sigma) antibodies. PhoN2-3xFLAG and PhoN2-6-His-tagged were detected by monoclonal anti-FLAG, and anti-His (Sigma) antibodies. PhoN2 was detected using mouse polyclonal antibodies [21]. Horseradish peroxidase-labeled goat anti-rabbit or anti-mouse antibodies were used as secondary antibodies and visualized by enhanced chemiluminescence (GE Healthcare). Measurement of the relative amounts of IcsA on intact bacteria was achieved essentially as previously described [36] as well as by immuno-dot blot analysis [37].

### Statistical analysis

Each experiment was repeated at least three times. Unless otherwise indicated, in indirect-immunofluorescence experiments, the fluorescence of 300 bacteria was counted for each sample (n = 300/experiment). In plaque assays, the size of 30 independent plaques was measured for each experiment (n = 30/experiment). Values are reported as means ± SD. Unpaired Student's *t* tests were used to determine statistical significance. P values ≤0.05 were considered to be statistically significant.

## Results

### Periplasmic PhoN2 polarly localizes at the poles of *S. flexneri* and *E. coli* cells

**Figure 1:**
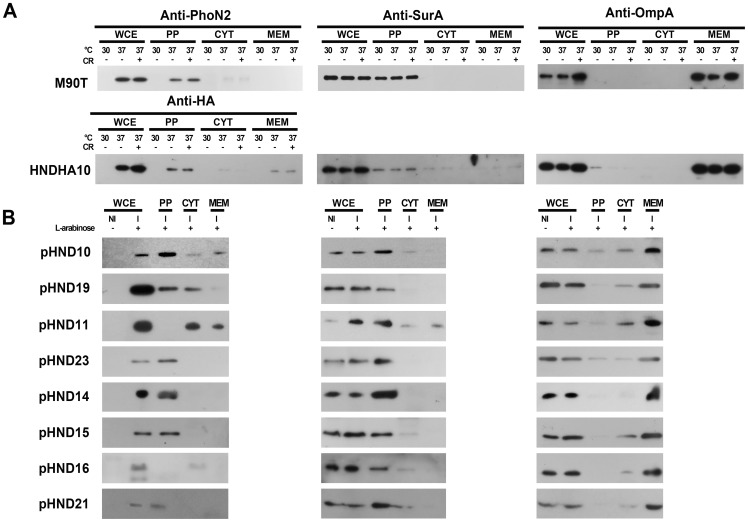
PhoN2 is a periplasmic protein. Exponentially-growing bacteria were harvested by centrifugation and supernatant, periplasmic and membrane fractions were prepared as described in Materials and Methods. Fractions were solubilised in Laemmli buffer and analyzed by Western blot analysis using polyclonal anti-PhoN2 [21], monoclonal anti-HA, polyclonal anti-SurA and anti-OmpA antibodies, as indicated. Panel A, bacterial fractions of wild-type *S. flexneri* strain M90T and of its mutant derivative strain HNDHA10 carrying the *phoN2*::HA fusion under the control of its natural promoter (Table S1). Panel B, bacterial fractions of the Δ*phoN2* mutant derivative strain HND115 complemented with plasmids pHND10, 19, 11, 23, 14, 15, 16 and 21 encoding the different HA-fused recombinant proteins under the control of an L-arabinose inducible promoter (Table S1). In these cases, 0.016% of L-arabinose was used to induce *phoN2*::HA expression. No specific signals of PhoN2-HA were evidenced when bacteria were grown in the absence of the inducer. Experiments were repeated at least three times and images are representative.

We have previously shown that PhoN2 (apyrase) is a virulence-associated factor of *S. flexneri* required for efficient cell-to-cell spreading [21]. By analyzing protein extracts of different bacterial compartments (periplasm, cytosol and membrane fractions; see Materials and Methods for details), of the wild-type *S. flexneri* strain M90T, grown at 30°C and at 37°C, in the presence or not of the Congo red dye, in order to induce *phoN2* MxiE-upregulated transcription [20], [21], [38], [39], we demonstrated that PhoN2 is a periplasmic protein as it has been previously suggested [24], [34]. Proteins were separated by SDS-PAGE and analyzed by Western blot using mouse polyclonal antibodies raised against PhoN2 [21]. To evaluate contaminations, the same protein extracts were also challenged with antibodies against SurA and OmpA, chosen as reporters of periplasmic and membrane proteins [40], [41], respectively. As shown in Figure 1A, PhoN2 was found to be almost exclusively associated with the periplasmic fraction of bacteria grown at 37°C in the presence or not of the inducer Congo red dye. Accordingly, the reporters SurA and OmpA were found to be almost exclusively associated with the periplasmic and membrane fractions, respectively. These results definitively demonstrated that PhoN2 is a periplasmic protein. As expected, *phoN2* expression was repressed when bacteria were grown at the non-permissive temperature of 30°C [20], [21].

To trace PhoN2 within bacterial cells and within bacteria inside infected HeLa cell monolayers, we constructed the mutant derivative strain HNDHA10 by allelic exchange [29] (see Materials and Methods for details). Strain HNDHA10 is a derivative of wild-type strain M90T carrying a C-terminal HA-tagged *phoN2* gene (*phoN2*::HA) (Table S1) so that the expression of the fused gene was under the control of its native promoter. The introduction of the HA-tag at the C-terminus of PhoN2 did influence neither its periplasmic delivery nor virulence expression since PhoN2-HA was predominantly detected within the periplasmic compartment (Fig. 1A), and HNDHA10 behaved as wild-type as far as synthesis and secretion of Ipa proteins, invasiveness, production of proper F-actin comet tails, protrusion and plaque formation are concerned (data not shown).

**Figure 2:**
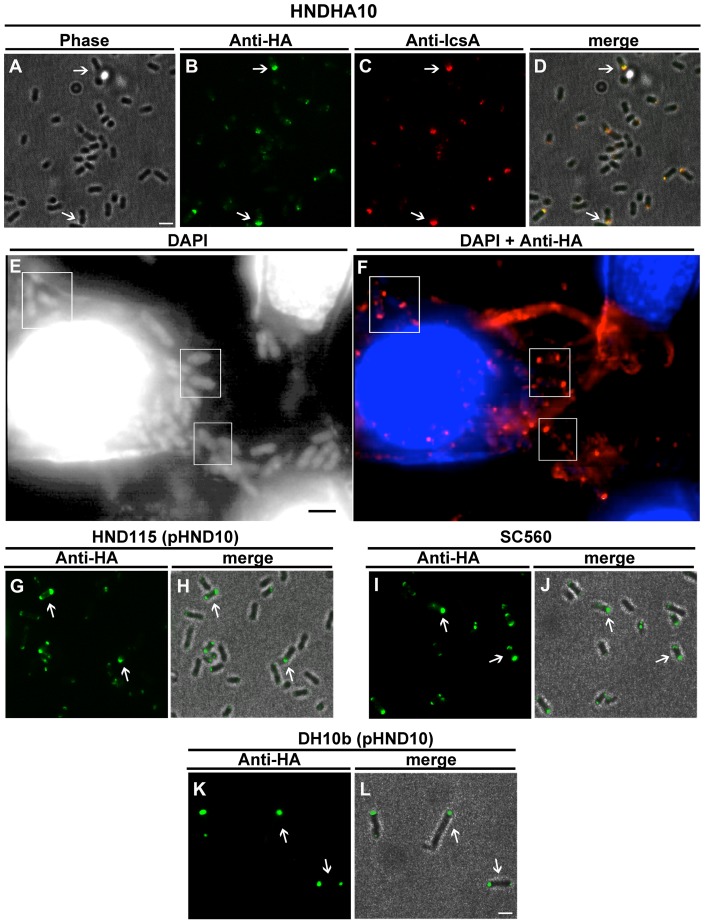
PhoN2-HA localizes at the poles of both exponentially-growing *S. flexneri* and of *E. coli* K-12 strains. Images are representative of phase-contrast, DAPI, anti-HA, anti-IcsA, and the merged fields. Panels A–D, HNDHA10 *phoN2*::HA. Panel A, phase-contrast; Panel B, bacteria stained with anti-HA (green); Panel C, bacteria stained with anti-IcsA (red); Panel D, overlay of fluorescence of panels A–C. Panels E–F, semi-confluent monolayers of HeLa cells were infected with exponentially-growing HNDHA10 *phoN2*::HA. After 2 h of infection, HeLa cells and bacteria inside cells were stained with DAPI (panel E) and with DAPI (blue) and anti-HA (red) (panel F, overlay of fluorescence). Squares indicate subpopulation of bacteria displaying polarly localized PhoN2-HA. Panels G–L, immunofluorescent staining of the Δ*phoN2* strain HND115, of the Δ*icsA* strain SC560 and of the *E. coli* K-12 strain DH10b (Table S1) complemented with plasmid pHND10 (panels G, I and K, anti-HA staining, green; panels H, J and L, overlay of the immunofluorescent images with the corresponding phase-contrast images). Bacteria shown in panels G–L were incubated in the presence of 0.016% of L-arabinose to induce *phoN2*::HA expression. No specific fluorescence signals were evidenced when bacteria were grown in the absence of the inducer (data not shown). Experiments were repeated at least three times and images are representative. Bars  = 2 µm.

Next, the intracellular localization of periplasmic PhoN2-HA was determined by indirect immunofluorescence experiments, using an anti-HA monoclonal antibody (Fig. 2A and B). Unexpectedly, periplasmic PhoN2-HA displayed polarly localized foci in HNDHA10. Fluorescence spots were detected at both bacterial poles in 54.8±5.8% and at only one pole in 39.3±4.4% of immunostained bacteria (n = 1.350; P<0.05). Remarkably, in bacteria displaying PhoN2-HA foci at both poles, we observed that the intensity of the fluorescence signal was significantly higher at what appeared to be the old pole (87.5±7.8%; *P*<0.05). Similar results were obtained when PhoN2 localization was measured in HeLa cell monolayers infected with HNDHA10 (Fig. 2E and F), indicating that up-regulation of *phoN2* expression, due to the activation of the TTS system [20], [21], [38], [39], had no effect on PhoN2 localization.

Since IcsA is a *Shigella* surface-exposed protein that localizes at the old bacterial pole where it mediates actin-based motility (ABM) [42], [43], the percentage of co-localization between IcsA and PhoN2-HA was measured by double immunofluorescent staining using anti-IcsA and anti-HA antibodies (see Materials and Methods for experimental details). As shown in Fig. 2C and D, IcsA and PhoN2-HA co-localized at the same pole in the vast majority of stained cells (92.5±4.6%), whereas only few bacteria (3.5±1.5%) displayed PhoN2 staining at the pole opposite to IcsA (n = 350; P<0.01). Furthermore, Western blot analysis of HNDHA10 whole cell extracts showed that PhoN2-HA was produced in equal amounts as the PhoN2 protein in wild-type, indicating that its polar localization was not due to PhoN2-HA overproduction (data not shown).

Thus, these results showed that the in the vast majority of HNDHA10 bacteria periplasmic PhoN2-HA foci were almost always found at the old bacterial pole just beneath IcsA. This result led us to infer that some interaction between the two proteins could occur.

To get further insight on the polar localization of PhoN2 and to evaluate possible PhoN2-IcsA interactions, recombinant plasmid pHND10, a pBAD28-based recombinant plasmid carrying the *phoN2*::HA fusion under the control of an L-arabinose-inducible promoter, was generated and introduced into HND115 and SC560, Δ*phoN2* and Δ*icsA* mutant derivatives of wild-type, respectively (Table S1). To induce *phoN2*::HA expression in HND115 (pHND10) at wild-type levels, Real-Time PCR analysis was performed using a wide range of L-arabinose concentrations (from 0.002 to 0.2%) and using the primer pair listed in Table S2. Results showed that 0.016% of L-arabinose was the concentration that induced a level of *phoN2*::HA expression which more closely resembled that occurring in the wild-type strain grown in the presence of the Congo red dye, *i.e.* in conditions of *phoN2* MxiE-up-regulated transcription (Figure S1). Accordingly, the concentration of 0.016% of L-arabinose was routinely used to induce *phoN2*::HA expression throughout the all study. As expected, the Δ*phoN2* mutant harboring pHND10, grown in the presence or not of 0.016% of L-arabinose, was as efficient as the wild-type strain in invading HeLa cell monolayers, while wild-type plaques were produced only when bacteria were grown in the presence of L-arabinose (data not shown and [21]). Immunofluorescence experiments showed that, as the strain HNDHA10, the Δ*phoN2* mutant harboring pHND10 displayed polarly localized foci both in exponentially-growing bacteria (91±6.5% of immunostained bacteria showed PhoN2-HA foci localized at both bacterial poles or at only one pole) (Fig. 2G and H) and within infected HeLa cells (data not shown). Moreover, Western blot analysis confirmed the periplasmic localization of the fused protein expressed by HND115 (pHND10) (Fig. 1B). No fluorescence signals were detected in the absence of the inducer L-arabinose (data not shown).

Indirect immunofluorescence experiments were carried out, using the Δ*icsA* mutant *S. flexneri* strain SC560 (Table S1) harbouring pHND10 grown in the presence of L-arabinose, to evaluate whether the presence of IcsA at the old bacterial pole was needed for the polar localization of PhoN2-HA. Since SC560 (pHND10) displayed PhoN2-HA polarly localized foci (Fig. 2I–J), we excluded that IcsA is required for the polar localization of PhoN2-HA. To confirm and extend these results, pHND10 was also introduced into the *E. coli* K-12 strain DH10b and again polar PhoN2-HA foci were visualized only when bacteria were grown in the presence of the inducer L-arabinose (Fig. 2K and L). These results indicated a common underlying mechanism for the delivery of PhoN2 to the poles of *S. flexneri* and *E. coli* and show that IcsA is not required for polar delivery of PhoN2.

### Characterization of PhoN2 domains involved in its polar localization

**Figure 3:**
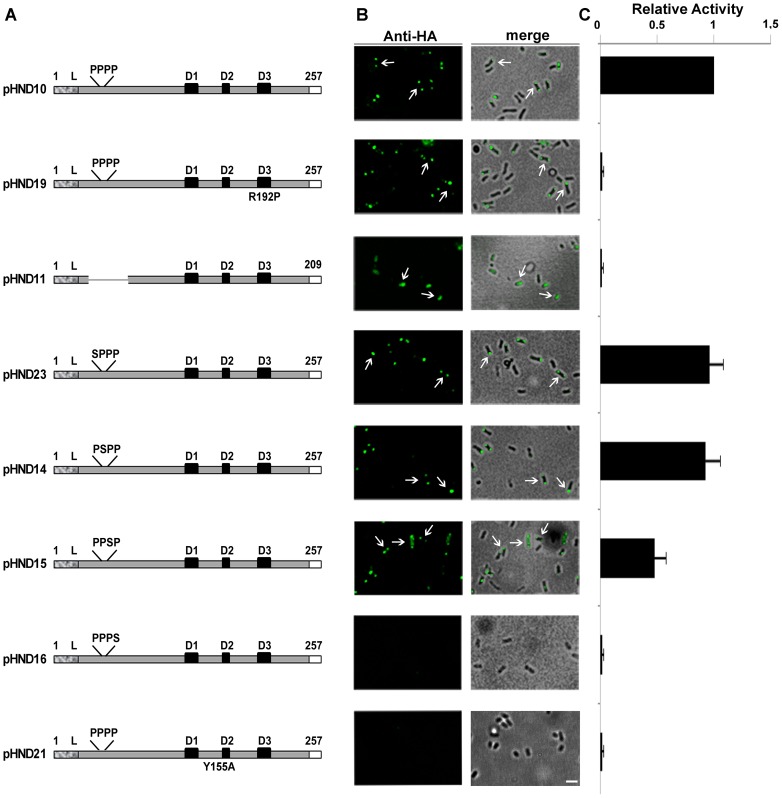
Determination of domains in PhoN2 required for polar localization and catalytic activity. Panel A, in-frame deletion and amino acid substitution mutants of the HA-tagged *phoN2* gene. Mutants were generated by PCR, as described in Materials and Methods, using pHND10 (Table S1) DNA as template and the primer pairs reported in Table S2. Amino acid residue 1 represents the first residue of the leader peptide (dotted box), while numbers on the right indicate the last residue of the tagged HA epitope (white box). The positions (not in scale) of the N-terminal polyproline PPPP motif, of the different P to S and of the Y to A amino acid substitutions are indicated. The conserved D1, D2 and D3 domains corresponding to the putative catalytic site of PhoN2 [24], [44] and the R192P substitution are indicated. Panel B, fluorescence microscopy and overlay of fluorescence with phase-contrast images of HND115 complemented with each of the PhoN2-HA recombinant plasmids listed in Panel A and grown in the presence of 0.016% of L-arabinose. Bacteria were labeled with anti-HA monoclonal antibody (green). Arrows indicate polar foci of PhoN2-HA. Panel C, units of ATP-hydrolyzing activity; one unit corresponds to 1 μmol of P_i_ min^−1^ mg^−1^ of protein; means ± standard deviations (error bars) of experiments that were performed more than three times. Images are representative. Bar  = 2 µm.

Functional and *in silico* analyses of PhoN2 have led to the identification of three relevant domains: i) an N-terminal 23 amino acid long leader sequence, which is likely responsible for the passage of PhoN2 across the cytoplasmic membrane; ii) a N-terminal domain encompassing an exposed ^43^PPPP^46^ sequence; and iii) a C-terminal wide domain encompassing the putative ATP-diphosphohydrolase catalytic site [24], [34], [44]. We have previously reported that while PhoN2 was required for proper *S. flexneri* cell-to-cell spread, its catalytic activity was dispensable [21]. To evaluate whether the catalytic activity of PhoN2 played a role in its polar localization, we introduced pHND19_R192P_ (a pHND10-derivative plasmid carrying the R192P amino acid substitution which has been shown to suppress apyrase activity [21], [44]) (Table S1) into the Δ*phoN2* mutant. Western blot analysis showed that the R192P-substituted protein was correctly delivered into the periplasmic compartment (Fig. 1B) and displayed polarly localized foci (Fig. 3), indicating that the deoxynucleotide triphosphate-hydrolyzing activity of PhoN2 is dispensable also for targeting PhoN2-HA to the bacterial poles. As expected, PhoN2-HA foci were detected only when bacteria were grown in the presence of L-arabinose (data not shown).

Next, an in frame deletion of the fused gene which removed a DNA region encompassing the encoded ^43^PPPP^46^ motif (nucleotides 79–223) was generated (plasmid pHND11_Δ79–223_; Table S1). When pHND11_Δ79–223_ was introduced into the Δ*phoN2* mutant, we found that although the recombinant protein was expressed at levels comparable to that expressed by pHND10 (Fig. S2), it was mainly found within the cytoplasmic compartment (Fig. 1B) accounting for the diffused fluorescence signals (Fig. 3B). The pHND11_Δ79–223_ mutant was partly visible in the membrane fraction (Fig. 1B). These results indicate that the delivery of the recombinant protein into the periplasm is impaired due to the lack of 27 to 75 amino acids, and suggest an improper protein folding that can lead to the formation of insoluble aggregates (Fig. 1B). These results indicated that the polyproline domain is important for both periplasmic delivery and polar localization of PhoN2. Remarkably, pHND11_Δ79–223_ failed to express apyrase activity (Fig. 3C), indicating that the polyproline domain also plays a role in catalysis.

To get further insight on the role of the polyproline motif on the periplasmic delivery of PhoN2-HA, on its polar localization and on the regulation of apyrase activity, four different derivatives of pHND10 carrying amino acid substitutions (P to S) of each of the P residues of the ^43^PPPP^46^ motif were constructed by using site-directed mutagenesis (see Materials and Methods for details). L-arabinose-induced Δ*phoN2* mutant carrying plasmids pHND23_SPPP_, pHND14_PSPP_, pHND15_PPSP_, and pHND16_PPPS_ (Table S1), were assayed for PhoN2-HA periplasmic delivery, polar localization and apyrase activity. As shown in Figures 1 and 3, substitution of the first (pHND23_SPPP_) and second (pHND14_PSPP_) proline residues neither influenced the periplasmic delivery nor the polar localization of the recombinant proteins (92.8±5.6% and 94.1±6.1% of fluorescent bacteria displayed PhoN2-HA polarly-localized foci, respectively) and both plasmids positively complemented the Δ*phoN2* mutant for apyrase activity. On the other hand, substitution of the third proline residue (pHND15_PPSP_), significantly affected its level of expression (Fig. S2) although apparently did not influence its periplasmic delivery (Fig. 1B). Moreover, while the percentage of bacteria presenting polarly-localized foci were significantly reduced (about 57.7±4.9 of fluorescent bacteria showed polar fluorescence spots, while 43.4±2.9% of bacteria showed a diffuse signal; *P*≤0.005), the mutant protein was still able to complement the Δ*phoN2* mutant for apyrase activity, although to a lower-extent when compared to pHND10, pHND23_SPPP_ and pHND14_PSPP_, (Fig. 3C). Remarkably, low-levels of PhoN2-HA expression (Fig. S2), loss of periplasmic delivery (Fig. 1B), absence of detectable fluorescence signals and inability to complement for apyrase activity (Fig. 3) was observed with the P to S substitution of the fourth proline residue (pHND16_PPPS_). These results were suggestive that the third (P45) and the fourth (P46) proline residues within the ^43^PPPP^46^ motif play a role on PhoN2 stability.

### The ^43^PPPP^46^ motif controls PhoN2 stability

**Figure 4:**
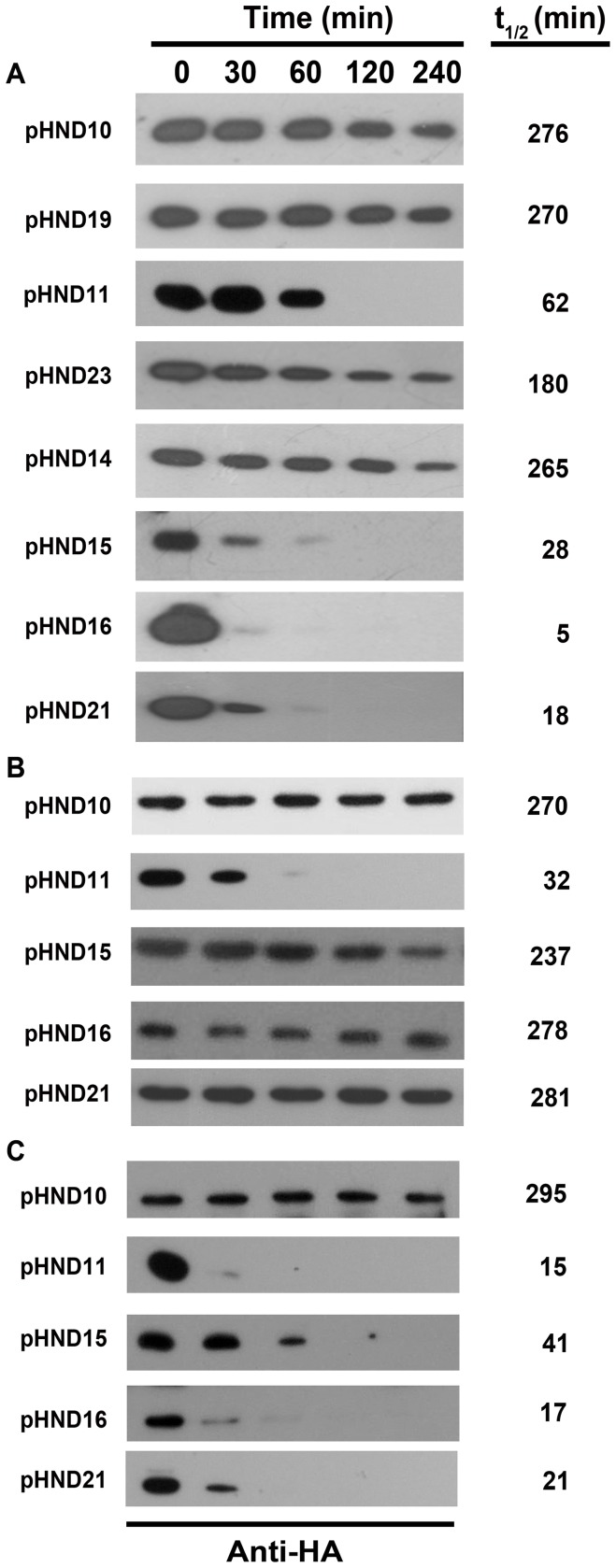
The polyproline PPPP motif and the Y155 residue are required for PhoN2 stability. The intracellular stability of recombinant proteins (Table S1) was determined in the *ΔphoN2* mutant strain HND115 complemented with the recombinant plasmids listed in Fig. 3 (Panel A). The relative stability of selected PhoN2-HA recombinant proteins (plasmids pHND10, pHND11, 15, 16 and 21) was evaluated in the *E. coli* K-12 strain DH10b (Panel B) and in BS176, a virulence plasmid-cured derivative of wild-type *S. flexneri* strain M90T (Table S1) (Panel C). Stability was measured after protein synthesis had been inhibited by the addition of 100 μg/ml of spectinomycin, as described in Materials and Methods. 0.016% of L-arabinose was used to induce *phoN2*::HA expression. Samples were removed at time points indicated at the top and whole cell extracts were analyzed by Western blot using anti-HA monoclonal antibody. Quantitative measurements were carried out and the calculated half-life of each recombinant protein is shown (right).

The finding that the P to S substituted recombinant proteins encoded by recombinant plasmids pHND15_PPSP_ and pHND14_PSPP_ were produced in very low amounts (Fig. S2) led us to evaluate PhoN2-HA protein stability of our mutants. Samples were removed at different time points after protein synthesis had been inhibited by the addition of 100 μg/ml of spectinomycin, and whole cell extracts were immunodetected by Western blot. Quantitative measurements were carried out to evaluate turnover properties of each recombinant protein. As shown in Figure 4A, the PhoN2-HA proteins encoded by L-arabinose-induced Δ*phoN2* mutant carrying pHND10 (control), pHND19_R192P_, pHND23_SPPP_, and pHND14_PSPP_ were stable throughout the duration of the experiment (half-lives of 276, 270, 180, and 265 min, respectively), while pHND11_Δ79–223_, pHND15_PPSP_, and pHND16_PPPS_ displayed a high degree of protein instability already 30 min after protein synthesis had been inhibited. We calculated half-lives of 62, 28 and 5 min for proteins encoded by pHND11_Δ79–223_, pHND15_PPSP_, and pHND16_PPPS_, respectively. These results showed that the deletion encompassing the ^43^PPPP^46^ motif and the P to S substitutions of the third and the fourth proline residues dramatically affect PhoN2 stability.

Next, to test if some specific protease was responsible for the degradation of these recombinant proteins, we evaluated their stability in different genetic backgrounds. Noteworthy, compared to pHND10, no significant difference in the steady-state levels of PhoN2-HA was noticed when recombinant plasmids pHND15_PPSP_, and pHND16_PPPS_ were introduced into the *E. coli* K-12 strain DH10b (half-lives of 270, 237 and 278 min, respectively) (Fig. 4B), while high levels of PhoN2 instability (half-lives of 15, 41 and 17 min) were detected when the same plasmids were introduced into strain BS176, a pINV-cured derivative of the *S. flexneri* strain M90T (Table S1) (Fig. 4C). These results indicated that the protease activity involved in the degradation of the recombinant proteins encoded by plasmids pHND15_PPSP_, and pHND16_PPPS_ is *S. flexneri*-specific and excluded that this activity could be encoded by gene(s) localized on the pINV of *S. flexneri* strain M90T. Remarkably, similar high levels of protein instability were detected both in *E. coli* DH10b and *S. flexneri* strain BS176 carrying plasmid pHND11_Δ79–223_ (32 and 15 min, respectively). These results indicated that the unfolded or misfolded protein encoded by pHND11_Δ79–223_ is degraded by different protease(s) present in both genetic backgrounds.

### The ^43^PPPP^46^ motif indirectly influences *S. flexneri* virulence

**Figure 5:**
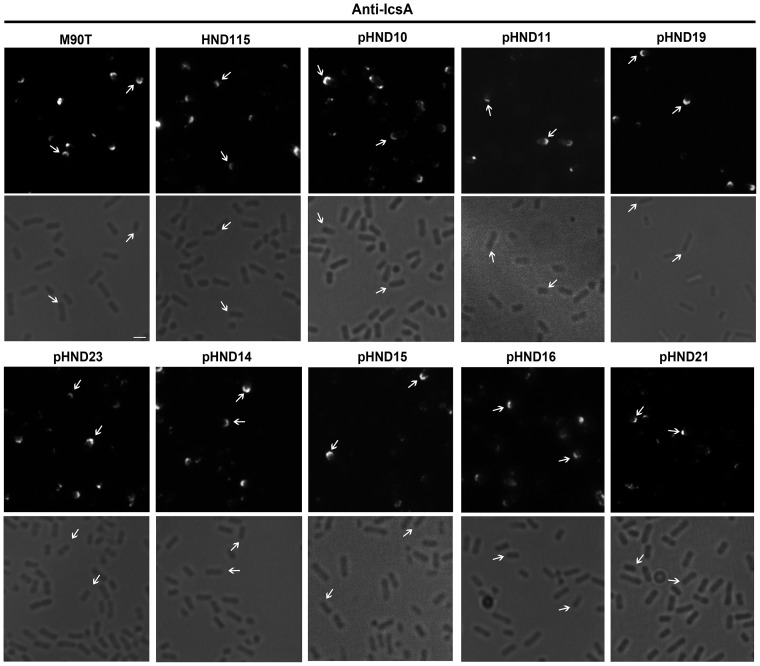
The ^43^PPPP^46^ motif is required for proper IcsA exposition. Bright fields and fluorescence microscopy of IcsA of the wild-type strain M90T, HND115 and HND115 complemented with recombinant plasmids pHND10, 11, 19, 23, 14, 15, 16 and 21 (indicated at the top). 0.016% of L-arabinose was used to induce *phoN2*::HA expression. IcsA was detected in exponentially-growing bacteria, fixed with paraformaldehyde and labelled with anti-IcsA rabbit polyclonal antibody. Subpopulations of IcsA-labelled bacteria are shown. Arrows, polar foci of IcsA. Experiments were repeated at least three times and typical results are shown. Bar  = 2 µm.

IcsA is the central bacterial mediator of ABM in *S. flexneri* [11]. Our previous results have shown that the lack of PhoN2 led to altered actin comet tails, consequently leading to inefficient cell-to cell spreading and to a small plaque phenotype [21]. To ascertain whether the lack of PhoN2 also influenced IcsA exposition, immunofluorescence experiments were performed. Indeed, compared to wild-type (88.02±6.3% of proper IcsA caps), the Δ*phoN2* mutant strain HND115 displayed a significant reduction of IcsA exposition (44.5±3.8%) (Fig. 5). This reduction was due to the lack of PhoN2, since complementation with pHND10 almost restored parental IcsA exposition (Fig. 5), only when bacteria were supplemented with L-arabinose (approximately 78.6±7.8% of immunostained complemented bacteria showed proper IcsA caps). These data were fully confirmed by quantitative surface immuno-dot blot analysis and immuno-assay, which together measured the levels of IcsA exposed on the surface of intact bacteria (Fig. S3A and B). Furthermore, we ruled out that the reduced IcsA exposition in the Δ*phoN2* mutant was due to reduced *icsA* transcription by Real-Time PCR analysis of cDNA preparations of the Δ*phoN2* mutant and of wild-type (data not shown). These results strongly indicated that PhoN2 plays a role in proper IcsA exposition likely by unmasking IcsA at the *S. flexneri* old bacterial pole.

As expected from the data of protein stability reported above, we found that, as pHND10 (control), pHND23_SPPP_, pHND14_PSPP_, pHND15_PPSP_ and pHND19_R192P_ were also able to positively complement IcsA exposition, while pHND16_PPPS_ did not (Fig. 5).

### Residue Y155 along with the ^43^PPPP^46^ motif is likely required for PhoN2 3-D structure

PhoN2 belongs to the family of the class A of the non-specific bacterial acid phosphatases (A-NSAPs) [17]. Among the relevant characteristics of this group of enzymes there is the presence of a N-terminal PPPP motif which was found to be highly conserved among all A-NSAPs analyzed so far. Crystallographic data of the A-NSAP PhoN from *Salmonella enterica* serovar Typhimurium indicated that the invariant residue Y154 stabilizes the loop harboring the active site residues by binding to the distal P40 residue of the PPPP motif [24], [26], [45].

To assess whether the corresponding Y residue in PhoN2 (residue Y155) could exert a similar structural role in its conformational stability, we introduced the Y155A amino acid substitution in pHND10, thus generating plasmid pHN21_Y155A_ (Table S1). Although the recombinant protein encoded by pHN21_Y155A_ was apparently delivered into the periplasmic compartment (although at lower levels, compared to pHND10; Fig. 1B), no fluorescence signal could be detected in immunofluorescence experiments and pHN21_Y155A_ failed to complement the Δ*phoN2* mutant for apyrase activity (Fig. 3B and C). Protein stability of the recombinant protein encoded by pHN21_Y155A_ was measured (Fig. 4A). Compared to pHND10 (half-life of 276 min), the Y155A substituted protein displayed a high degree of instability (half-live of 18 min) and, as the recombinant proteins encoded by plasmids pHND15_PPSP_ and pHND16_PPPS_, its stability was strictly dependent on the same unknown *S. flexneri*-specific protease(s) (half-live of 281 min in *E. coli* and 21 min in *S. flexneri*; Fig. 4B and C). Taken together, these results, although indirectly, indicate that, similarly to PhoN of *S. typhimurium*, the Y155 of PhoN2 contributes to 3-D structure, likely by binding the distal P residues (P45 and/or P46) of PhoN2 (Fig. S4).

### PhoN2 binds to the OM protein A (OmpA)

To find specific interactor(s) that could account for the polar localization of PhoN2, co-purification experiments were conducted with 3xFLAG- and 6-His-C-terminal-tagged PhoN2, using affinity chromatography resins. Bound resins, challenged with clear lysates of the wild-type strain, failed to detect any interaction partner (data not shown). Interaction partners were then searched by the two-hybrid technique in yeast. By using pGBKT7/*phoN2* as bait, a cDNA library of the wild-type strain, fused in frame to the coding sequence of the GAL4-activating domain of plasmid pGADT7-Rec (Table S1), was screened as detailed in Materials and Methods. *Saccharomyces cerevisiae* AH109 competent cells were simultaneously transformed with the bait plasmid and the prey plasmid library and spread onto selective plates. Plasmid DNA preparations of 16 independent transformants grown on minimal medium agar plates lacking leucine, tryptophan, histidine and adenine and expressing high-level of α-galactosidase, were used to transform *E. coli* DH10b competent cells selecting for kanamycin-resistance. The DNA inserts carried by independent prey plasmid DNA preparations were sequenced. Remarkably, the great majority of proteins encoded by the prey plasmids (13 out of the 16 inserts examined) corresponded to the C-terminal domain of OmpA (Table 1). Moreover, all OmpA inserts encompassed a common region composed of residues 189–273. The 189–273 residues represent the C-terminal domain of OmpA known to be exposed into the bacterial periplasm when the protein is in the 8-stranded β-barrel conformation [46], [47]. These results were indicative of a PhoN2-OmpA periplasmic interaction involving the periplasmic-exposed C-terminal domain of OmpA. This interaction was not detected in co-purification experiments (see above), probably because the soluble form of OmpA was either not present in the bacterial clear lysate or in an amount not sufficient to be visualized by Coomassie staining.

The sequence of the inserts of the three remaining prey plasmids (Table 1) encompassed amino acid residues of three different cytosolic *S. flexneri* proteins, namely Adh, AspRS and EF-2. The inserts encoded by these three plasmids were not analyzed further.

**Figure 6:**
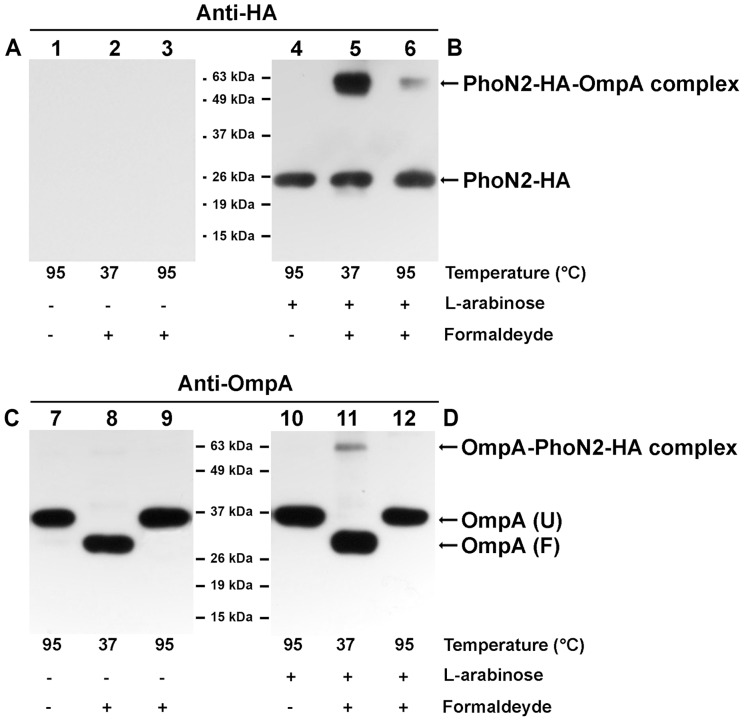
*In vivo* cross-linking experiments. The ^43^PPPP^46^ motif of PhoN2 is not required for the PhoN2-OmpA interaction. Cross-linking of HND115 (pHND10) was achieved by treating bacteria with formaldehyde to a final concentration of 1%, as described in Materials and Methods. Samples were suspended in Laemmli buffer and either heated at 37°C for 10 min to maintain cross-links or at 95°C for 20 min to break cross-links. Equal amounts of proteins were analyzed by Western blot. A protein molecular weight marker (Pierce) was used to determine the molecular weight of proteins. Immunoblotting was carried out with monoclonal anti-HA (Panels A and B) or polyclonal anti-OmpA antibodies (Panels C and D). Expression of PhoN2-HA was achieved by growing bacteria in the presence of 0.016% of L-arabinose. Panels A and C, bacteria not induced with L-arabinose; Panels B and D, L-arabinose induced bacteria. OmpA (U), unfolded OmpA; OmpA (F), folded OmpA [48]. Experiments were repeated at least three times and typical results are shown.

To confirm the interaction between PhoN2 and OmpA, cross-linking experiments were performed, as detailed in Materials and Methods, (Fig. 6). Cross-linking was performed with the *ΔphoN2* mutant carrying pHND10, grown in the presence (Fig. 6B and D) or not (Fig. 6A and C) of L-arabinose. Total protein extracts were separated by SDS-PAGE and immunoblotted using anti-HA monoclonal (Fig. 6A and B) and anti-OmpA polyclonal antibodies (Fig. 6C and D). Immunoblot of bacterial extracts, denatured at 95°C in Laemmli buffer prior to electrophoresis, detected PhoN2-HA (Fig. 6B, lanes 4 and 6) and unfolded OmpA (Fig. 6C and D, lanes 7, 9, 10 and 12) of the expected molecular size (28 and 35 kDa, respectively), while in samples heated at 37°C OmpA migrates as a folded 30 kDa protein (Fig. 6C and D, lanes 8 and 11) [48]. On the other hand, extracts of formaldehyde-treated bacteria, expressing both PhoN2-HA and OmpA, generated an additional molecular species only when samples were heated at 37°C (Fig. 6B and D, lanes 5 and 11). This new molecular species migrated with an apparent mass of 63 kDa, consistent with the molecular size corresponding to the sum of mature PhoN2-HA and OmpA. The intensity of the new band was strongly reduced when heated at 95°C (Fig. 6B and D, lanes 6 and 12). As expected, no PhoN2-HA signal was detected in the absence of L-arabinose (Fig. 6A, lanes 1 to 3). These results definitely confirmed a PhoN2-OmpA specific interaction.

### The ^43^PPPP^46^ motif is not involved in the PhoN2-OmpA interaction

An important class of protein-interaction domains is constituted by protein modules recognizing exposed proline-rich motifs [49]. Cross-linking experiments were performed to investigate whether the ^43^PPPP^46^ motif plays any role in PhoN2-OmpA interaction. Extracts of formaldehyde-treated bacteria, grown in the presence or not of 0.016% of L-arabinose, were separated by SDS-PAGE and immunoblotted, as described above. As expected, recombinant proteins encoded by pHND10 (control), pHND23_SPPP_, pHND14_PSPP_ and pHND19_R192P_ displayed the expected 63 kDa band in the extracts heated at 37°C whose intensity was greatly reduced when extracts were heated at 95°C. Noteworthy, recombinant proteins encoded by plasmids pHND15_PPSP_ and pHND11_Δ79–223_, although presenting high-levels of protein instability (half-lives of 28 and 62 min, respectively; Figure 4A), still displayed the expected 63 kDa band whose intensity was considerably lower than that of pHND10 (data not shown). On the other hand pHND16_PPPS_, which encodes the more unstable recombinant protein, failed to display any sign of a specific interaction (data not shown). These results ruled out that the ^43^PPPP^46^ motif could be involved in the PhoN2-OmpA interaction.

### OmpA is not required for polar localization of PhoN2

**Figure 7:**
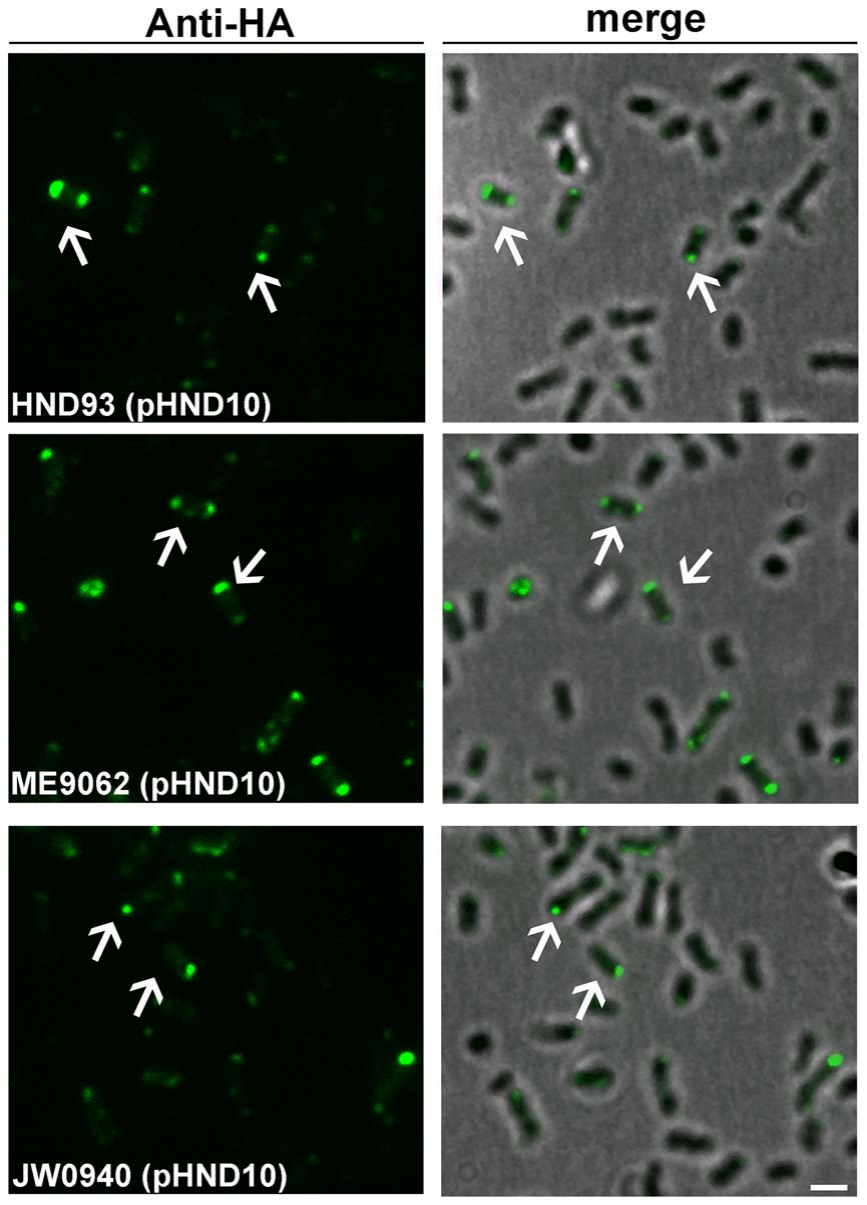
OmpA is not required in the process of polar localization of PhoN2-HA. Fluorescence microscopy of the *S. flexneri* mutant strain HND93 (Δ*phoN2* Δ*ompA*), of the *E. coli* strain ME9062 (*ompA* ^+^) and of its Δ*ompA* derivative strain JW0940 (Table S1) complemented with plasmid pHND10. Fluorescence microscopy and overlay of fluorescence with phase contrast images are shown. Bacteria were grown in the presence of 0.016% of L-arabinose to induce *phoN2*::HA expression and labeled with anti-HA monoclonal antibody. Panel A, HND93 (pHND10); Panel B, ME9062 (pHND10); Panel C, JW0940 (pHND10). Arrows, polar foci of PhoN2-HA. Experiments were repeated at least three times and typical results are shown. Bar  = 2 µm.

To investigate whether the polar localization of PhoN2 was due to the interaction with OmpA, wild-type derivative strains HND93 Δ*phoN2* Δ*ompA* (Table S1) was generated. Remarkably, HND93 complemented with plasmid pHND10, displayed polarly localized PhoN2-HA foci (Fig. 7) in 92.3±5.2% in immunostained bacteria (n = 290). Similar results were obtained when the HND92 Δ*ompA* strain (Table S1) was complemented with plasmid pHND10 (data not shown). Furthermore, both *ompA*-proficient and -deficient *E. coli* K-12 strains ME9062 and JW0940 (Table S1) complemented with plasmid pHND10 displayed polarly localized PhoN2-HA foci (Fig. 7). These results ruled out that the PhoN2-HA-OmpA interaction is required for the polar localization of PhoN2-HA.

### PhoN2-OmpA interaction: a computational analysis

When PhoN2 sequence was threaded into the available 3D structure of the highly similar (73% sequence identity) *Escherichia blattae* acid phosphatase EBPase, it was predicted to have a highly overlapping 3-D structure [24]. According to the inferred putative PhoN2 structure, a long unstructured N-terminal region folds onto the protein core mainly by the interaction of the ^43^PPPP^46^ motif with the Y155 residue. Molecular docking simulations of the ^183^PAPAP^187^ motif of OmpA onto the PhoN2 protein core led us to hypothesize that the OmpA-PhoN2 complex might result from the swapping of the PhoN2 ^43^PPPP^46^ motif with the OmpA ^183^PAPAP^187^ motif. To test this prediction, we introduced P to A substitutions into the ^183^PAPAP^187^ motif of the *ompA* gene, thus generating plasmid pAAAOmpA encoding the ^183^AAAAA^187^ motif (Table S1). Mutated OmpA behaved as native OmpA, for what it concerned localization within the membrane fraction and presentation of the expected heat-variable mobility (data not shown, [50], [51]). Therefore, HND93 *ompA phoN2* was co-transformed with plasmids pHND10 and pOmpA or pAAAOmpA (Table S1) to perform in vivo cross-linking experiments. No difference in the cross-linked complex was observed between the formaldehyde-treated whole cell extracts from HND93 (pHND10 pAAAOmpA) (Fig. S5B and D) and from the positive control strain HND93 (pHND10 pOmpA) (Fig. S5A and C). The same protein complex was also displayed by the Δ*phoN2* mutant harboring plasmid pHND21_Y155A_ or pHND10 used as a positive control (data not shown). No signal corresponding to PhoN2-HA could be detected in the absence of L-arabinose (data not shown). These results seemed to rule out that the ^183^PAPAP^187^ motif and the Y155 residue could be involved in the PhoN2-HA-OmpA interaction. However, we cannot exclude that other motifs, together with ^183^PAPAP^187^ and the Y155 residue, might concur in this interaction.

## Discussion

We have previously shown that apyrase (PhoN2) of *S. flexneri* is required, in a deoxynucleotide triphosphate-hydrolyzing activity-independent manner, for ABM and proper plaque formation [19], [21]. The data presented in this work corroborate and extend our knowledge on apyrase. Here we definitively confirmed that PhoN2 is a periplasmic protein (Fig. 1A). Furthermore, by substituting the pINV-encoded *phoN2* gene of the wild-type *S. flexneri* M90T strain with a C-terminal HA-tagged *phoN2* gene, thus under the control of its natural promoter, or by using *phoN2*::HA cloned into a plasmid vector under the control of an L-arabinose inducible promoter, we show that PhoN2-HA strongly accumulates at bacterial poles (Fig. 2A–H), with the great majority of bacteria presenting PhoN2 in correspondence of the old pole, where IcsA is exposed. Noteworthy, the polar delivery of PhoN2-HA was also observed in *S. flexneri* bacteria inside HeLa cells (Fig. 2E–F) as well as in a *E. coli* K-12 strain complemented with *phoN2*::HA (plasmid pHND10; Table S1) (Fig. 2K–L), indicating that PhoN2 polar delivery is conserved among the two bacterial species, that is independent from the T3S system-mediated up-regulation of *phoN2*::HA expression and from the expression of other pINV-encoded genes.

Moreover, although here we show that IcsA was not required for the polar localization of PhoN2-HA (Fig. 2I–J), we show that PhoN2 is required for proper IcsA exposition since, compared to wild-type, the Δ*phoN2* mutant displayed a significant reduction of IcsA exposition (smaller IcsA caps), and complementation with plasmid pHND10 restored proper IcsA exposition (Figs. 5 and S3). The observed reduction of IcsA exposition detected in the Δ*phoN2* mutant was neither due to reduced *icsA* expression [21] nor to a difference in membrane fluidity, as determined by treating the wild-type and the Δ*phoN2* mutant either with chlorpromazine (a drug which has been shown to decrease membrane fluidity in *E. coli*; [52], [53]) or with Triton X-100 (a detergent we used to partially unmask IcsA at bacterial surface) (Scribano D., unpublished results).

IcsA is a key virulence determinant of *S. flexneri* being the exclusive factor required for ABM [3], [9], [11]. Although the mechanism responsible for the polar localization of IcsA has not been fully elucidated yet, the current model of delivery of IcsA on the bacterial surface indicates that, following its secretion through the Sec translocon, IcsA directly inserts into the OM at the old bacterial pole by the aid of specific proteins that recognize and bind IcsA [14], [42], [53], [54], [55], [56]. Subsequently, it has been proposed that membrane-bound IcsA diffuses toward the new pole by membrane fluidity, forming a gradient that decreases in concentration with distance from the old pole. Although at a low efficiency, IcsP is thought to be the protease involved in the sharpening of the IcsA gradient leading to the release of the 95 kDa N-terminal fragment IcsA* and to the exclusive exposition of IcsA at the old bacterial pole [52], [53], [57], [58], [59], [60]. On the other hand, it has also been hypothesized that IcsA exposition is likely masked by LPS molecules, probably in combination with OM proteins, at lateral bacterial surfaces in order to hamper its function at those sites [61], [62]. Moreover, it has also been suggested that the polar unmasking of IcsA at the old bacterial pole could be due to the presence of LPS molecules of shorter length at this site in order to ensure proper IcsA exposition and function [61]. Furthermore, it has been also suggested that OM proteins could cooperate to allow proper polar IcsA exposition and function [61], [62].

Next, in order to identify PhoN2-HA domains involved in its polar localization, deletion and amino acid substitutions were generated in the coding sequence of *phoN2*:HA. As relevant features, mature periplasmic PhoN2 displays an exposed polyproline motif (^43^PPPP^46^) at its N-terminal domain, which is highly conserved among the A-NSAP protein family, and a C-terminal domain encompassing the putative ATP-diphosphohydrolase catalytic site [17], [21], [22], [24], [25], [44]. By introducing plasmid pHND19_R192P_ (Table S1) into the Δ*phoN2* mutant, we ruled out that the catalytic activity of apyrase is required for its polar localization (Fig. 3). Furthermore, proline-rich sequences were found to be critical for both intra- and inter-molecular interactions [49]. Therefore, we focused our attention on the N-terminal ^43^PPPP^46^ motif. That the ^43^PPPP^46^ motif played a role in the polar localization of PhoN2 was evidenced by the observation that a deletion encompassing this motif (plasmid pHND11_Δ79–223_; Table S1) led to a recombinant protein unable to be exported into the periplasmic compartment, to form polarly-localized foci and to express its catalytic activity (Figs. 1 and 3). To better define the role ^43^PPPP^46^ motif in the polar delivery of PhoN2-HA as well as in its periplasmic export and apyrase activity, we generated, by site-directed mutagenesis, P to S substitutions of each of the four proline residues (Table S1). The results obtained showed that the third (P45S; pHND15_PPSP_) and the fourth (P46S; pHND16_PPPS_) proline residues exerted a key role on the correct folding of PhoN2-HA, affecting protein stability, periplasmic delivery, polar localization, IcsA exposition and apyrase catalytic activity (Figs. 1, 3, 4 and 5). Interestingly, we observed a graduation of protein stability and function dependent on individual P to S substitutions introduced into PhoN2-HA (Fig. 4A). The high protein instability showed by P45S (pHND15_PPSP_) and P46S (pHND15_PPSP_) was likely due to the generation of unfolded or misfolded proteins whose conformational state probably allows exposure of buried cleavage sites for proteolysis. Unfolded or misfolded proteins can be degraded by a variety of bacterial proteases. The observation that the P45S and P46S recombinant proteins were stable when expressed in a *E. coli* K-12 genetic background while rapid degradation occurred when expressed in the pINV-cured *S. flexneri* strain BS176 (Fig. 4B and C) indicated that these recombinant proteins are recognized and degraded by some *S. flexneri-*specific protease, whose structural gene is not localized on the pINV. Interestingly, the recombinant protein expressed by plasmid pHND11_Δ79–223_ showed an high degree of protein instability both in *S. flexneri* and *E. coli* (Fig. 4B and C), indicating that in this case protein degradation was not due to the action of the *S. flexneri*-specific protease. Comparative sequence analysis (NCBI) of the currently available genome of *S. flexneri* M90T [63] with that of the *E. coli* K-12 strain MG1655 revealed the presence in the *S. flexneri* genome of genes encoding for protease activity that are absent in *E. coli* K-12 chromosome. Experiments are underway to identify and characterize this *S. flexneri*-specific protease.

The finding that the ^43^PPPP^46^ motif is important for protein folding and stability of PhoN2 is supported by previous studies showing that the conserved N-terminal PPPP motif plays a key role on the conformational stability of members of the A-NSAPs protein family. In this context, although crystal structures are available neither for PhoN2 nor for PiACP, an A-NSAP of *Prevotella intermedia* most closely related to PhoN2 [17], [25], [34], [64], it has been hypothesized that the planar exposed PPPP motif of the two proteins ensures proper orientation and angle to the putative catalytic pocket, thus playing a structural role in the expression of their catalytic activity [24], [25]. Moreover, crystallographic analysis of the PhoN, an A-NSAP from *Salmonella typhimurium*, and of EB-NSAP, an A-NSAP from *Escherichia blattae*, indicated that amino acid substitutions within the PPPP motif highly influence the conformational stability (and thus the catalytic activity) of these two proteins. The loss of the conformational stability was shown to be likely due to the loss of an hydrogen bond between the PPPP stretch and an invariant Y residue (Y155 in PhoN2) [26], [45]. These findings prompted us to investigate the role of the Y155 residue of PhoN2-HA in protein stability. As expected, the introduction of plasmid pHND21_Y155A_ (Table S1) into the Δ*phoN2* led to the production of a Y155A recombinant protein displaying a high degree of instability and the unfolded or misfolded protein was found to be degraded by the same *S. flexneri*-specific protease described above (Fig. 4). Moreover, despite its high instability, a small protein fraction was found to be correctly delivered into the periplasmic compartment, although the Y155A recombinant protein did not display polarly localized foci, was unable to restore the formation of proper IcsA caps and failed to express apyrase catalytic activity (Figs. 1, 3 and 5). These results indicate that the Y155 residue by binding to the ^43^PPPP^46^ motif (likely to the P45 and/or P46 residues) concurs to the conformational stability of PhoN2. Thus, the interaction between the PPPP motif and the invariant Y residue appears to be a conserved feature among the A-NSAPs proteins family.

Next, the mechanism underlying the polar localization of PhoN2 was investigated by searching for *S. flexneri* proteins which may interact with PhoN2. A two-hybrid screen in yeast and *in vivo* cross-linking experiments showed that PhoN2 binds to the periplasmic exposed C-terminal domain of the OM protein OmpA (Table 1 and Fig. 6). Unexpectedly, in spite of the PhoN2-OmpA interaction, OmpA was not required for the polar localization of PhoN2 (Fig. 7A–C), indicating that probably other proteins may be involved in this phenomenon.

OmpA is a multifaceted, extremely abundant protein (about 100,000 molecules/cell) associated, in cooperation with other factors, to the virulence of several bacterial pathogens [47]. Analysis of detailed high-resolution structural data demonstrated that OmpA is a pore that may adopt alternative conformations: i) an 8-stranded β-barrel small pore with a large C-terminal domain exposed into the periplasm, perhaps interacting with peptidoglycan or with other unknown periplasmic components; and ii) a predominant 16-stranded β-barrel large pore, involving additional eight β-strands from the C-terminal domain [47], [65], [66], [67]. It has been proposed that the large pore conformation may be the native fully folded form of the protein, while the small pore variant represents an exceptionally stable folding intermediate [47], [66], [67].

The finding that periplasmic polarly localized PhoN2 binds OmpA to its periplasmic exposed C-terminal domain (Table 1) and the notion that this periplasmic exposed domain exists only when OmpA is in the eight-stranded β-barrel conformation [46], [47], leads to hypothesize that this binding may well contribute to stabilize bound OmpA molecules in a stable 8-stranded β-barrel folding intermediate at the bacterial poles.

Finally, a visual inspection of the model structure of PhoN2 and OmpA was carried out in order to identify domains involved in the PhoN2-OmpA interaction. The computational analysis predicted that the periplasmic exposed C-terminal proline-rich ^183^PAPAP^187^ motif of OmpA could be involved in this interaction by displacement of the N-terminal region of PhoN2, and by interaction with the protein region surrounding Y155 residue of PhoN2. To evaluate this prediction, *in vivo* cross-linking experiments were carried out. The results obtained ruled out that the ^183^PAPAP^187^ motif and the Y155 residue are involved in the PhoN2-OmpA interaction (Fig. S5). We cannot exclude that other motifs, together with the ^183^PAPAP^187^ motif and the Y155 residue, might concur to the formation of this interaction.

**Figure 8:**
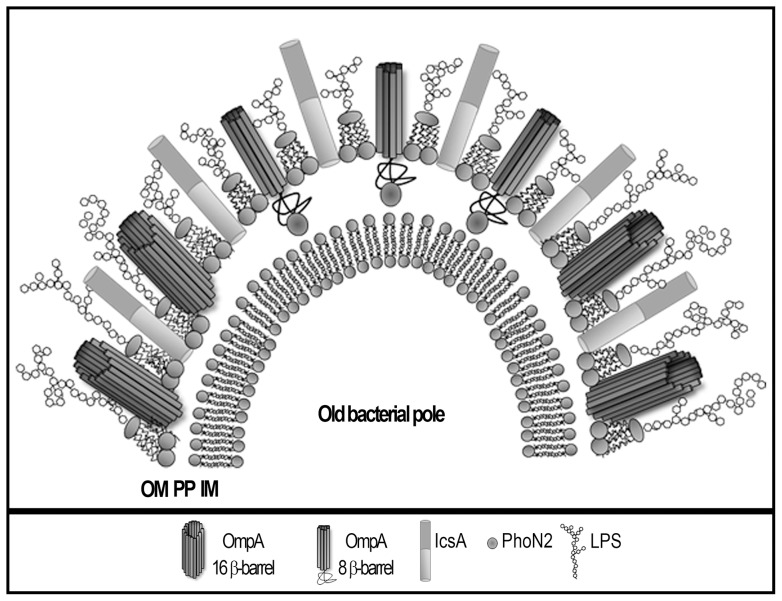
Model of the role of periplasmic PhoN2 in *S. flexneri* in proper IcsA exposition. Polarly localized PhoN2, by binding the C-terminal periplasmic domain of OmpA only at the old bacterial pole, stabilizes OmpA in the small pore conformation thereby enabling proper IcsA exposition and function.

In conclusion, our results demonstrate that PhoN2 is the first example of a periplasmic polarly localized virulence-associated protein of *S. flexneri* whose interaction with OmpA might concur to proper IcsA exposition and function. In this respect, we would like to speculate that the stabilization of OmpA in the 8-stranded β-barrel stable folding intermediate, due to PhoN2 binding to the C-terminal periplasmic exposed domain of OmpA, likely together with the presence of LPS molecules of shorter length at the old bacterial pole [61], [62], may favor proper IcsA exposition and function only at this site (Fig. 8). In this respect, we have recently shown that OmpA (likely in its 16-stranded β-barrel large pore conformation) strictly masks IcsA exposition and function at bacterial lateral surfaces [37].

On the other hand, when PhoN2 is lacking, OmpA is no more stabilized in the small pore folding intermediate at the bacterial pole thus leading to transformation of OmpA into predominant large pore conformational molecules whose presence at the old bacterial pole, by itself or in combination with other cellular components, likely reduce the extent of IcsA exposition and function at this site.

Future investigation is needed to unravel the mechanism underlining the polar delivery of PhoN2 in the bacterial periplasm and to determine the nature of the interaction between PhoN2 and OmpA.

## Supporting Information

Figure S1 **Real-time quantitative PCR analysis.** Relative expression of the *phoN2* gene in wild-type *S. flexneri* strain M90T (grown in the presence or not of 0.01% of the Congo red dye) and of *phoN2*::HA in the Δ*phoN2* mutant strain HND115 complemented with plasmid pHND10 (grown in the presence of different L-arabinose concentrations). Total RNA was extracted from exponentially-growing bacteria (OD_600_ = 0.8). Histograms show *phoN2* (grey bars) and *phoN2*::HA (black bars) expression relative to that of the *nusA* gene of wild-type M90T [32]. CT values were normalized to levels of *nusA* RNA to correct for variations in bacterial numbers. Results shown are means and standard deviations from triplicate experiments (P<0.05).
(TIF)

Figure S2 **The P to S substitution of the third and fourth proline residue in the ^43^PPPP^46^ motif affects expression of the recombinant proteins.** Whole cell extracts of exponentially-growing Δ*phoN2* mutant strain HND115 harboring the different recombinant plasmids pHND10, 11, 19, 23, 14, 15, 16 and 21 (indicated at the top) were solubilised in Laemmli buffer and analyzed in Western blot using monoclonal anti-HA antibody. Bacteria were grown in the presence of 0.016% of L-arabinose to induce *phoN2*::HA expression. Panel A, recombinant PhoN2-HA proteins encoded by plasmids pHND10, 11, 19, 23, 14 and 21; Panel B, lane cutting and pasting were needed to visualize the protein signals recombinant PhoN2-HA proteins encoded by pHND15 and 16, which required different exposure times. A protein molecular weight marker (Pierce) was used to determine the molecular weight of proteins.
(TIF)

Figure S3 **The lack of PhoN2 influenced IcsA exposition.** Quantitative surface immunodetection of IcsA: (Panel A) dot blots of intact bacteria probed with anti-IcsA antibody; and (Panel B) intact bacteria treated with anti-IcsA and anti-*S. flexneri* antibodies. The amount of antibody bound to the bacterial surface was determined by labeling with HRP-conjugated secondary antibody and measuring the HRP enzymatic activity (A_370_). Means and standard deviation of three independent experiments are shown. Blots are representative. Arrow indicates IcsA.
(TIF)

Figure S4 **Schematic representation of the PhoN2 structural model showing the molecular environment of Y155.** The backbone of the long unstructured N-terminal region is shown in purple. Note the location of Y155 between the N-terminal L42, P45, P46, A205 hydrophobic residues and the strong hydrogen bond (yellow dashed line; donor-acceptor distance ∼2.7 Å) between Y155 hydroxyl group and P43 carbonyl group.
(TIF)

Figure S5 **The ^183^PAPAP^187^ motif of OmpA is not required for the PhoN2-OmpA interaction.** *In vivo* cross-linking experiments. Cross-linking of the *S. flexneri* mutant strain HND93, complemented either with plasmids pHND10 and pOmpA (Panels A and C), or with plasmids pHND10 and pAAAOmpA (Panels B and D, Table S1) was achieved by treating bacteria with formaldehyde to a final concentration of 1%, as described in Materials and Methods. Samples were suspended in Laemmli buffer and either heated at 37°C for 10 min to maintain cross-links or at 95°C for 20 min to break cross-links. Equal amounts of proteins were analyzed by Western blot. A protein molecular weight marker (Pierce) was used to determine the molecular weight of proteins. Immunoblotting was carried out using monoclonal anti-HA (Panels A and B) or polyclonal anti-OmpA antibodies (Panels C and D). Expression of *phoN2*-HA was achieved by growing bacteria in the presence of 0.016% of L-arabinose. OmpA (U), unfolded OmpA; OmpA (F), folded OmpA [48]. The relative position of the PhoN2-HA-OmpA complex is indicated (right). Experiments were repeated at least three times and typical results are shown.
(TIF)

Table S1 **Bacterial/yeast strains and plasmids used in this work.**
(DOC)

Table S2 **Primers used in this work.**
(DOCX)

## References

[1] Rokney A, Shagan M, Kessel M, Smith Y, Rosenshine I, et al (2009) *E. coli* transports aggregated proteins to the poles by a specific and energy-dependent process. J Mol Biol 392: 589–601. 19596340 10.1016/j.jmb.2009.07.009

[2] Henderson IR, Navarro-Garcia F, Desvaux M, Fernandez RC, Ala'Aldeen D (2004) Type V protein secretion pathway: the autotransporter story. Microbiol Mol Biol Rev 68: 692–744. 15590781 10.1128/MMBR.68.4.692-744.2004 PMC539010

[3] Jain S, van Ulsen P, Benz I, Schmidt MA, Fernandez R, et al (2006) Polar lcalization of the autotransporter family of large bacterial virulence factors. J Bacteriol 188: 4841–4850. 16788193 10.1128/JB.00326-06 PMC1483012

[4] Leo JC, Grin I, Linke D (2012) Type V secretion: mechanism(s) of autotransport through the bacterial outer membrane. Philos Trans R Soc Lond B Biol Sci. 367: 1088–1101. 10.1098/rstb.2011.0208 PMC3297439 22411980

[5] Ieva R, Bernstein D (2009) Interaction of an autotransporter passenger domain with BamA during its translocation across the bacterial outer membrane. Proc Natl Acad Sci USA 106: 19120–19125. 19850876 10.1073/pnas.0907912106 PMC2776426

[6] Knowles TJ, Scott-Tucker A, Overduin M, Henderson IR (2009) Membrane protein architects: the role of the BAM complex in outer membrane protein assembly. Nat Rev Microbiol 7: 206–214. 19182809 10.1038/nrmicro2069

[7] Cornelis GR (2006) The type III secretion injectisome. Nat Rev Microbiol 4: 811–825. 17041629 10.1038/nrmicro1526

[8] Cossart P, Sansonetti PJ (2006) Bacterial invasion: the paradigms of enteroinvasive pathogens. Science 304: 242–248. 10.1126/science.1090124 15073367

[9] Bernardini ML, Mounier J, d'Hauteville H, Coquis-Rondon M, Sansonetti PJ (1989) Identification of *icsA*, a plasmid locus of *Shigella flexneri* that governs bacterial intra- and intercellular spread through interaction with F-actin. Proc Natl Acad Sci USA 86: 3867–3871. 2542950 10.1073/pnas.86.10.3867 PMC287242

[10] Buchrieser C, Glaser P, Rusniok C, Nedjari H, d'Hauteville H, et al (2000) The virulence plasmid pWR100 and the repertoire of proteins secreted by the type III secretion apparatus of *Shigella flexneri* . Mol Microbiol 38: 760–771. 11115111 10.1046/j.1365-2958.2000.02179.x

[11] Schroeder GN, Hilbi H (2008) Molecular pathogenesis of *Shigella* spp.: controlling host cell signaling, invasion, and death by type III secretion. Clin Microbiol Rev 21: 134–156. 18202440 10.1128/CMR.00032-07 PMC2223840

[12] Suzuki T, Miki H, Takenawa T, Sasakawa C (1998) Neural Wiskott-Aldrich syndrome protein is implicated in the actin-based motility of *Shigella flexneri* . EMBO J 17: 2767–2776. 9582270 10.1093/emboj/17.10.2767 PMC1170617

[13] Cossart P (2000) Actin-based motility of pathogens: the Arp2/3 complex is a central player. Cell Microbiol 2: 195–205. 11207576 10.1046/j.1462-5822.2000.00053.x

[14] Charles M, Pérez M, Kobil JH, Goldberg MB (2001) Polar targeting of *Shigella* virulence factor IcsA in *Enterobacteriacae* and *Vibrio*. Proc Natl Acad Sci U S A. 98: 9871–9876. 10.1073/pnas.171310498 PMC55545 11481451

[15] Goldberg MB (2001) Actin-based motility of intracellular microbial pathogens. Microbiol Mol Biol Rev 65: 595–626. 11729265 10.1128/MMBR.65.4.595-626.2001 PMC99042

[16] May KL, Morona R (2008) Mutagenesis of the *Shigella flexneri* autotransporter IcsA reveals novel functional regions involved in IcsA biogenesis and recruitment of host neural Wiscott-Aldrich syndrome protein. J Bacteriol 190: 4666–4676. 18456802 10.1128/JB.00093-08 PMC2446779

[17] Rossolini GM, Schippa S, Riccio ML, Berlutti F, Macaskie LE, et al (1998) Bacterial nonspecific acid phosphohydrolases: physiology, evolution and use as tools in microbial biotechnology. Cell Mol Life Sci 54: 833–850. 9760992 10.1007/s000180050212 PMC11147330

[18] Komoszyński M, Wojtczak A (1996) Apyrase (ATP diphosphohydrolase, EC 3.6.1.5): function and relationship with ATPases. Biochim Biophys Acta 1310: 233–241. 8611638 10.1016/0167-4889(95)00135-2

[19] Santapaola D, Casalino M, Petrucca A, Presutti C, Zagaglia C, et al (2002) Enteroinvasive *Escherichia coli* virulence plasmid-carried apyrase (*apy*) and *ospB* genes are organized as a bicistronic operon and are subjected to differential expression. Microbiology 148: 2519–2529. 12177345 10.1099/00221287-148-8-2519

[20] Le Gall T, Mavris M, Martino MC, Bernardini ML, Denamur E, et al (2005) Analysis of virulence plasmid gene expression defines three classes of effectors in the type III secretion system of *Shigella flexneri* . Microbiology 151: 951–962. 15758240 10.1099/mic.0.27639-0

[21] Santapaola D, Del Chierico F, Petrucca A, Uzzau S, Casalino M, et al (2006) Apyrase, the product of the virulence plasmid-encoded *phoN2* (*apy*) gene of *Shigella flexneri*, is necessary for proper unipolar IcsA localization and for efficient intercellular spread. J Bacteriol 188: 1620–1627. 16452446 10.1128/JB.188.4.1620-1627.2006 PMC1367242

[22] Niebuhr K, Ebel F, Frank R, Reinhard M, Domann E, et al (1997) A novel proline-rich motif present in ActA of *Listeria monocytogenes* and cytoskeletal proteins is the ligand for the EVH1 domain, a protein module present in the Ena/VASp family. EMBO J 16: 5433–5444. 9312002 10.1093/emboj/16.17.5433 PMC1170174

[23] Suzuki T, Sasakawa C (2001) Molecular basis of the intracellular spreading of *Shigella* . Infect Immun 69: 5959–5966. 11553531 10.1128/IAI.69.10.5959-5966.2001 PMC98722

[24] Babu MM, Kalamalakkannan S, Subrahmanyam YVBK, Sankaran K (2002) *Shigella* apyrase-a novel variant of bacterial acid phosphatases? FEBS Lett 512: 8–12. 11852042 10.1016/s0014-5793(02)02287-1

[25] Ansai T, Chen X, Barik S, Takehara T (2002) Conserved proline residues near the N-terminus are important for enzymatic activity of class A bacterial acid phosphatases. Arch Biochem Biophys 408: 144–146. 12485613 10.1016/s0003-9861(02)00524-6

[26] Makde RD, Mahajan SK, Kumar V (2007) Structure and mutational analysis of the PhoN protein of *Salmonella typhimurium* provide insight into mechanistic details. Biochemistry 46: 2079–2090. 17263560 10.1021/bi062180g

[27] Miller JH (1972) Experiments in molecular genetics, p. 252–255. Cold Spring Harbor Laboratory, Cold Spring Harbor, N.Y.

[28] Sambrook J, Russel DW (2001) Molecular cloning: a laboratory manual, 3^rd^ ed. Cold Spring.

[29] Uzzau S, Figueroa-Bossi N, Rubino S, Bossi L (2001) Epitome tagging of chromosomal genes in *Salmonella* . Proc Natl Acad Sci USA 98: 15264–15269. 11742086 10.1073/pnas.261348198 PMC65018

[30] Guzman LM, Belin D, Carson MJ, Beckwith J (1995) Tight regulation, modulation, and high-level expression by vectors containing the arabinose P_BAD_ promoter. J Bacteriol 177: 4121–4130. 7608087 10.1128/jb.177.14.4121-4130.1995 PMC177145

[31] von Gabain A, Belasco JG, Schottel JL, Chang AC, Cohen SN (1983) Decay of mRNA in *Escherichia coli*: investigation of the fate of specific segments of transcripts. Proc. Natl Acad Sci USA 80: 653–657. 10.1073/pnas.80.3.653 PMC393437 6187001

[32] Di Martino ML, Fioravanti R, Barbabella G, Prosseda G, Colonna B, et al. (2013) Molecular evolution of the nicotinic acid requirement within the *Shigella*/EIEC pathotype. Int J Med Microbiol. 13. doi:pii: S1438-4221(13)00145-8. 10.1016/j.ijmm.2013.09.007. 10.1016/j.ijmm.2013.09.007 24120364

[33] Laemmli UK (1970) Cleavage of structural proteins during the assembly of the head of bacteriophage T4. Nature 227: 680–685. 5432063 10.1038/227680a0

[34] Bhargava T, Datta S, Ramakrishnan V, Roy RK, Sankaran K, et al (1995) Virulent *Shigella* codes for a soluble apyrase: identification, characterization and cloning of the gene. Curr Sci 68: 293–300.

[35] Berlutti F, Casalino M, Zagaglia C, Fradiani PA, Visca P, et al (1998) Expression of the virulence plasmid-carried apyrase gene (*apy*) of enteroinvasive *Escherichia coli* and *Shigella flexneri* is under the control of H-NS and the VirF and VirB regulatory cascade. Infect Immun 66: 4957–4964. 9746603 10.1128/iai.66.10.4957-4964.1998 PMC108614

[36] Purdy GE, Fisher CR, Payne SM (2007) IcsA surface presentation in *Shigella flexneri* requires the periplasmic chaperones DegP, Skp, and SurA. J Bacteriol 189: 5566–5573. 17526712 10.1128/JB.00483-07 PMC1951818

[37] Ambrosi C, Pompili M, Scribano D, Zagaglia C, Ripa S, et al (2012) Outer membrane protein A (OmpA): a new player in *Shigella flexneri* protrusion formation and inter-cellular spreading. PLoS One (7) e49625. 10.1371/journal.pone.0049625 PMC3498225 23166731

[38] Menard R, Sansonetti P, Parsot C (1994) The secretion of the *Shigella flexneri* Ipa invasins is activated by epithelial cells and controlled by IpaB and IpaD. Embo J 13: 5293–5302. 7957095 10.1002/j.1460-2075.1994.tb06863.x PMC395485

[39] Bahrani FK, Sansonetti PJ, Parsot C (1997) Secretion of Ipa proteins by *Shigella flexneri*: inducer molecules and kinetics of activation. Infect Immun 65: 4005–4010. 9316999 10.1128/iai.65.10.4005-4010.1997 PMC175575

[40] Lazar SW, Kolter R (1996) SurA assists the folding of *Escherichia coli* outer membrane proteins. J Bacteriol 178: 1770–1773. 8626309 10.1128/jb.178.6.1770-1773.1996 PMC177866

[41] Krishnan S, Prasadarao NV (2012) Outer membrane protein A and OprF: versatile roles in Gram-negative bacterial infections. FEBS J. doi:10.1111/j.1742-4658.2012.08482.x. 10.1111/j.1742-4658.2012.08482.x PMC3338869 22240162

[42] Goldberg MB, Barzu O, Parsot C, Sansonetti PJ (1993) Unipolar localization and ATPase activity of IcsA, a *Shigella flexneri* protein involved in intracellular movement. J Bacteriol 175: 2189–2196. 8468279 10.1128/jb.175.8.2189-2196.1993 PMC204503

[43] Goldberg MB, Theriot JA, Sansonetti PJ (1994) Regulation of surface presentation of IcsA, a *Shigella* protein essential to intracellular movement and spread, is growth phase dependent. Infect Immun 62: 5664–5668. 7960150 10.1128/iai.62.12.5664-5668.1994 PMC303317

[44] Sarli S, Nicoletti M, Schippa S, Del Chierico F, Santapaola D, et al (2005) Ala160 and His116 residues are involved in activity and specificity of apyrase, an ATP-hydrolyzing enzyme produced by enteroinvasive *Escherichia coli* . Microbiology 151: 2853–2860. 16151198 10.1099/mic.0.28142-0

[45] Ishikawa K, Mihara Y, Gondoh K, Suzuki E, Asano Y (2000) X-ray structures of a novel acid phosphatase from *Escherichia blattae* and its complex with the transition-state analog molybdate. EMBO J 19: 2412–2423. 10835340 10.1093/emboj/19.11.2412 PMC212741

[46] Koebnik R, Kramer L (1995) Membrane assembly of circularly permuted variants of the *E. coli* outer membrane protein OmpA. J Mol Biol 250: 617–626. 7623380 10.1006/jmbi.1995.0403

[47] Smith SGJ, Mahon V, Lambert MA, Fagan RP (2007) A molecular Swiss army knife: OmpA structure, function and expression. FEMS Microbiol Lett 273: 1–11. 17559395 10.1111/j.1574-6968.2007.00778.x

[48] Schweizer M, Hindennach I, Garten W, Henning U (1978) Major proteins of the *Escherichia coli* outer cell envelope membrane. Interaction of protein II with lipopolysaccharide. Eur J Biochem 82: 211–217. 340230 10.1111/j.1432-1033.1978.tb12013.x

[49] Kay BK, Williamson MP, Sudol M (2000) The importance of being proline: the interaction of proline-rich motifs in signaling proteins with their cognate domains. FASEB J 14: 231–241. 10657980

[50] Cock H, van Blokland S, Tommassen J (1996) In vitro insertion and assembly of outer membrane protein PhoE of *Escherichia coli* K-12 into the outer membrane. Role of Triton X-100. J Biol Chem 271: 12885–12890. 8662743 10.1074/jbc.271.22.12885

[51] Sugawara E, Steiert M, Rouhani S, Nikaido H (1996) Secondary structure of the outer membrane proteins OmpA of *Escherichia coli* and OprF of *Pseudomonas aeruginosa* . J Bacteriol 178: 6067–6069. 8830709 10.1128/jb.178.20.6067-6069.1996 PMC178469

[52] Tanji K, Ohta Y, Kawato S, Mizushima T, Natori S, et al (1992) Decrease by psychotropic drugs and local anaesthetics of membrane fluidity measured by fluorescence anisotropy in *E scherichia coli* . J Pharm Pharmacol 44: 1036–1037. 1361554

[53] Robbins JR, Monack D, McCallum SJ, Vegas A, Pham E, et al (2001) The making of a gradient: IcsA (VirG) polarity in *Shigella flexneri* . Mol Microbiol 41: 861–872. 11532149 10.1046/j.1365-2958.2001.02552.x

[54] Brandon LD, Goehring N, Janakiraman A, Yan AW, Wu T, et al (2003) IcsA, a polarly localized autotransporter with an atypical signal peptide, uses the Sec apparatus for secretion, although the Sec apparatus is circumferentially distributed. Mol Microbiol 50: 45–60. 14507362 10.1046/j.1365-2958.2003.03674.x

[55] Wagner JK, Heindl JE, Gray AN, Jain S, Goldberg MB (2009) Contribution of the periplasmic chaperone Skp to efficient presentation of the autotransporter IcsA on the surface of *Shigella flexneri* . J Bacteriol 191: 815–821. 19047350 10.1128/JB.00989-08 PMC2632083

[56] Tran CN, Giangrossi M, Prosseda G, Brandi A, Di Martino ML, et al (2011) A multifactor regulatory circuit involving H-NS, VirF and an antisense RNA modulates transcription of the virulence gene *icsA* of *Shigella flexneri* . Nucleic Acids Res 39: 8122–8134. 21724612 10.1093/nar/gkr521 PMC3185424

[57] d'Hauteville H, Dufourcq-Lagelouse R, Nato F, Sansonetti PJ (1996) Lack of cleavage of IcsA in *Shigella flexneri* causes aberrant movement and allow demonstration of a cross-reactive eukaryotic protein. Infect Immun 64: 511–517. 8550200 10.1128/iai.64.2.511-517.1996 PMC173794

[58] Egile C, d'Hauteville H, Parsot C, Sansonetti PJ (1997) SopA, the outer membrane protease responsuble for polar localization of IcsA in *Shigella flexneri* . Mol Microbiol 23: 1063–1073. 9076742 10.1046/j.1365-2958.1997.2871652.x

[59] Shere KD, Sallustio S, Manessis A, D'Aversa TG, Goldberg MB (1997) Disruption of IcsP, the major *Shigella* protease that cleaves IcsA, accelerates actin-based motility. Mol Microbiol 25: 451–462. 9302008 10.1046/j.1365-2958.1997.4681827.x

[60] Steinhauer J, Agha R, Pham T, Varga AW, Goldberg MB (1999) The unipolar *Shigella* surface protein IcsA is targeted directly to the bacterial old pole: IcsP cleavage of IcsA occurs over the entire bacterial surface. Mol Microbiol 32: 367–377. 10231492 10.1046/j.1365-2958.1999.01356.x

[61] Morona R, Daniels C, Van Den Bosh L (2003) Genetic modulation of *Shigella flexneri* 2a lipopolysaccharide O antigen modal chain length reveals that it has been optimized for virulence. Microbiology 149: 925–939. 12686635 10.1099/mic.0.26141-0

[62] Morona R, Van Den Bosch L (2003) Lipopolysaccharide O antigen chains mask IcsA (VirG) in *Shigella flexneri* . FEMS Microbiol Lett 221: 173–180. 12725923 10.1016/S0378-1097(03)00210-6

[63] Onodera NT, Ryu J, Durbic T, Nislow C, Archibald JM, et al (2012) Genome sequence of *Shigella flexneri* serotype 5a strain M90T Sm. J. Bacteriol 194: 3022. 22582379 10.1128/JB.00393-12 PMC3370606

[64] Chen X, Ansai T, Awano S, Iida T, Barik S, et al (1999) Isolation, cloning, and expression of an acid phosphatase containing phosphotyrosyl phosphatase activity from *Prevotella intermedia* . J Bacteriol 181: 7107–7114. 10559178 10.1128/jb.181.22.7107-7114.1999 PMC94187

[65] Zakharian E, Reusch RN (2003) Outer membrane protein A of *Escherichia coli* forms temperature-sensitive channels in planar lipid bilayers. FEBS Lett 555: 229–235. 14644420 10.1016/s0014-5793(03)01236-5

[66] Zakharian E, Reusch RN (2005) Kinetics of folding of *Escherichia coli* OmpA from narrow to large pore conformation in a planar bilayer. Biochemistry 44: 6701–6707. 15850404 10.1021/bi047278e

[67] Khalid S, Bond PJ, Carpenter T, Sansom MS (2008) OmpA: gating and dynamics via molecular dynamics simulations. Biochim Biophys Acta 1778: 1871–1880. 17601489 10.1016/j.bbamem.2007.05.024

