# Supplementary figures and images for: Polar Localization of PhoN2, a Periplasmic Virulence-Associated Factor of Shigella flexneri, Is Required for Proper IcsA Exposition at the Old Bacterial Pole
Source: PLoS One. 2014 Feb 27;9(2):e90230. doi: 10.1371/journal.pone.0090230 (PMC3937361; doi:10.1371/journal.pone.0090230)

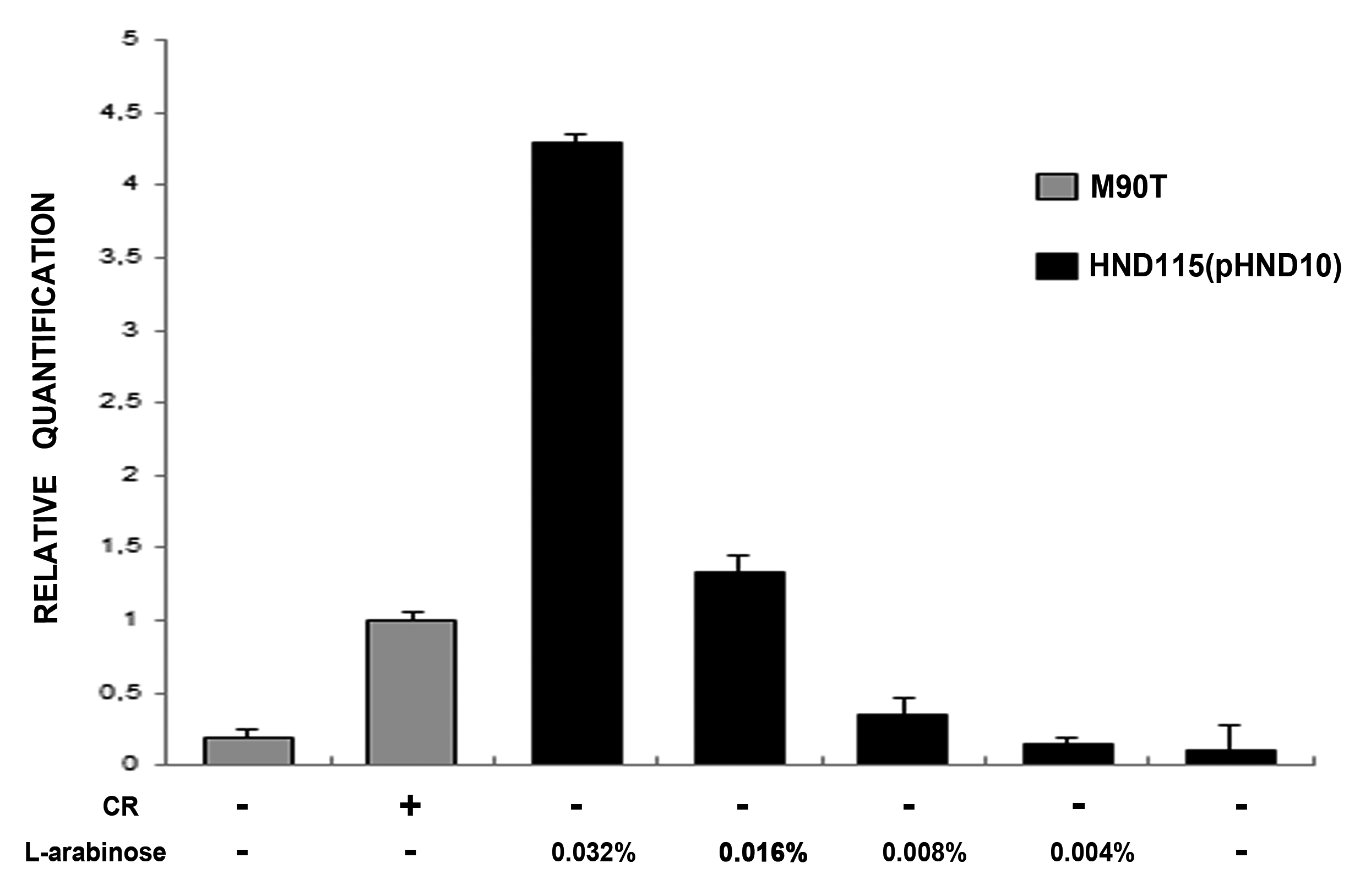

Supplement: Figure S1 — Real-time quantitative PCR analysis. Relative expression of the phoN2 gene in wild-type S. flexneri strain M90T (grown in the presence or not of 0.01% of the Congo red dye) and of phoN2::HA in the ΔphoN2 mutant strain HND115 complemented with plasmid pHND10 (grown in the presence of different L-arabinose concentrations). Total RNA was extracted from exponentially-growing bacteria (OD600 = 0.8). Histograms show phoN2 (grey bars) and phoN2::HA (black bars) expression relative to that of the nusA gene of wild-type M90T [32]. CT values were normalized to levels of nusA RNA to correct for variations in bacterial numbers. Results shown are means and standard deviations from triplicate experiments (P<0.05). (TIF) [file pone.0090230.s001.tif]

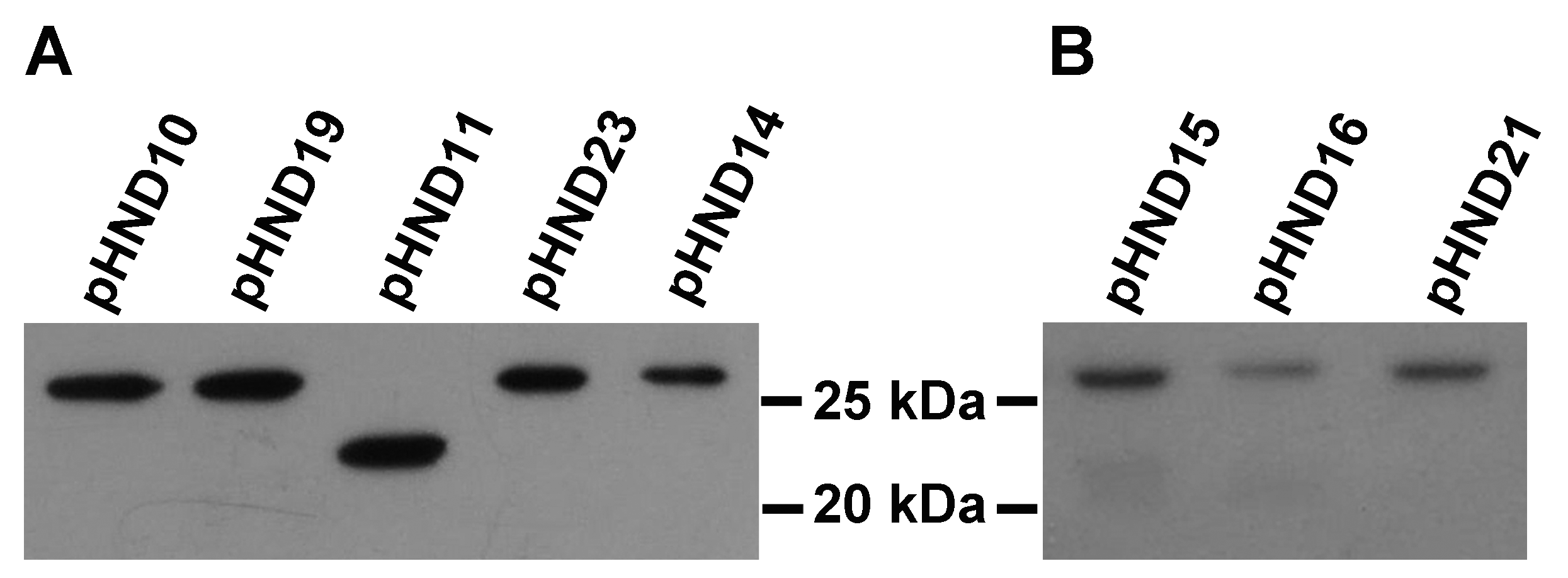

Supplement: Figure S2 — The P to S substitution of the third and fourth proline residue in the 43PPPP46 motif affects expression of the recombinant proteins. Whole cell extracts of exponentially-growing ΔphoN2 mutant strain HND115 harboring the different recombinant plasmids pHND10, 11, 19, 23, 14, 15, 16 and 21 (indicated at the top) were solubilised in Laemmli buffer and analyzed in Western blot using monoclonal anti-HA antibody. Bacteria were grown in the presence of 0.016% of L-arabinose to induce phoN2::HA expression. Panel A, recombinant PhoN2-HA proteins encoded by plasmids pHND10, 11, 19, 23, 14 and 21; Panel B, lane cutting and pasting were needed to visualize the protein signals recombinant PhoN2-HA proteins encoded by pHND15 and 16, which required different exposure times. A protein molecular weight marker (Pierce) was used to determine the molecular weight of proteins. (TIF) [file pone.0090230.s002.tif]

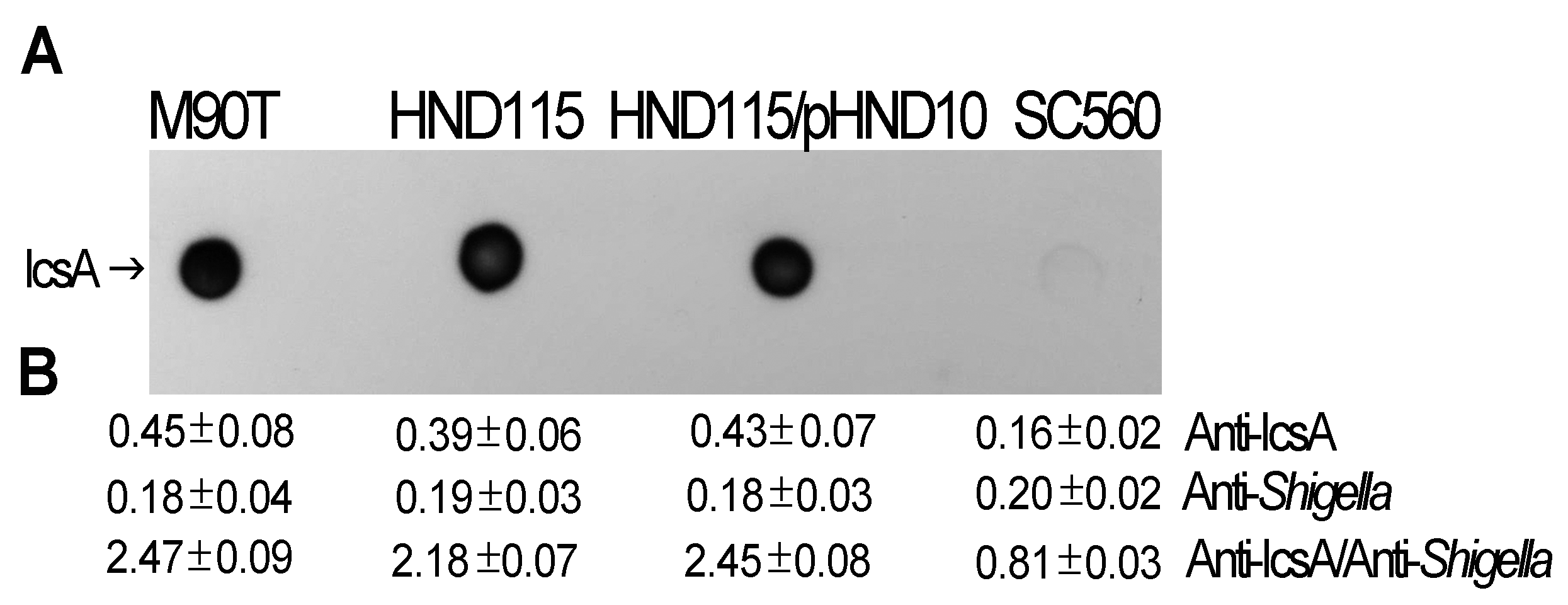

Supplement: Figure S3 — The lack of PhoN2 influenced IcsA exposition. Quantitative surface immunodetection of IcsA: (Panel A) dot blots of intact bacteria probed with anti-IcsA antibody; and (Panel B) intact bacteria treated with anti-IcsA and anti-S. flexneri antibodies. The amount of antibody bound to the bacterial surface was determined by labeling with HRP-conjugated secondary antibody and measuring the HRP enzymatic activity (A370). Means and standard deviation of three independent experiments are shown. Blots are representative. Arrow indicates IcsA. (TIF) [file pone.0090230.s003.tif]

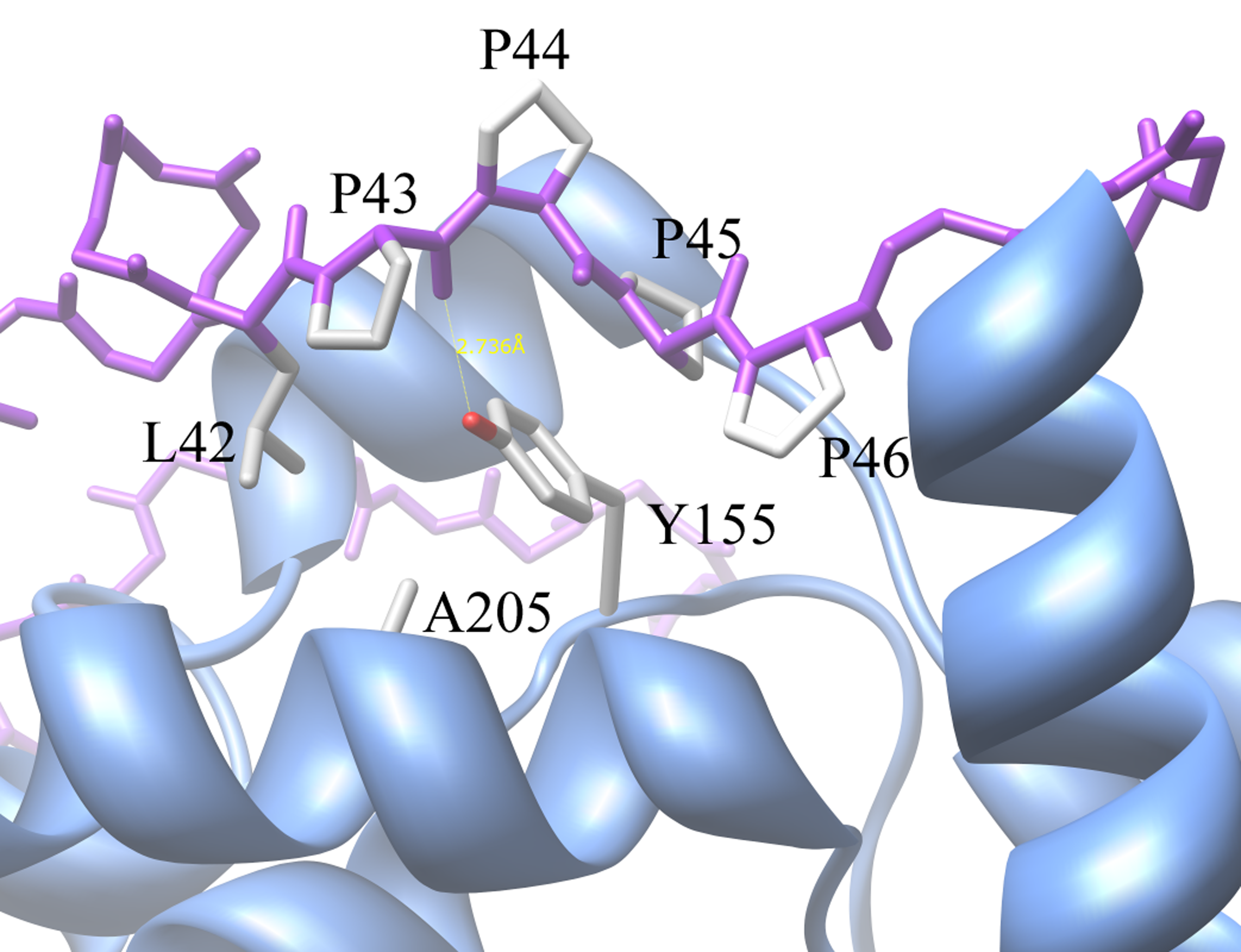

Supplement: Figure S4 — Schematic representation of the PhoN2 structural model showing the molecular environment of Y155. The backbone of the long unstructured N-terminal region is shown in purple. Note the location of Y155 between the N-terminal L42, P45, P46, A205 hydrophobic residues and the strong hydrogen bond (yellow dashed line; donor-acceptor distance ∼2.7 Å) between Y155 hydroxyl group and P43 carbonyl group. (TIF) [file pone.0090230.s004.tif]

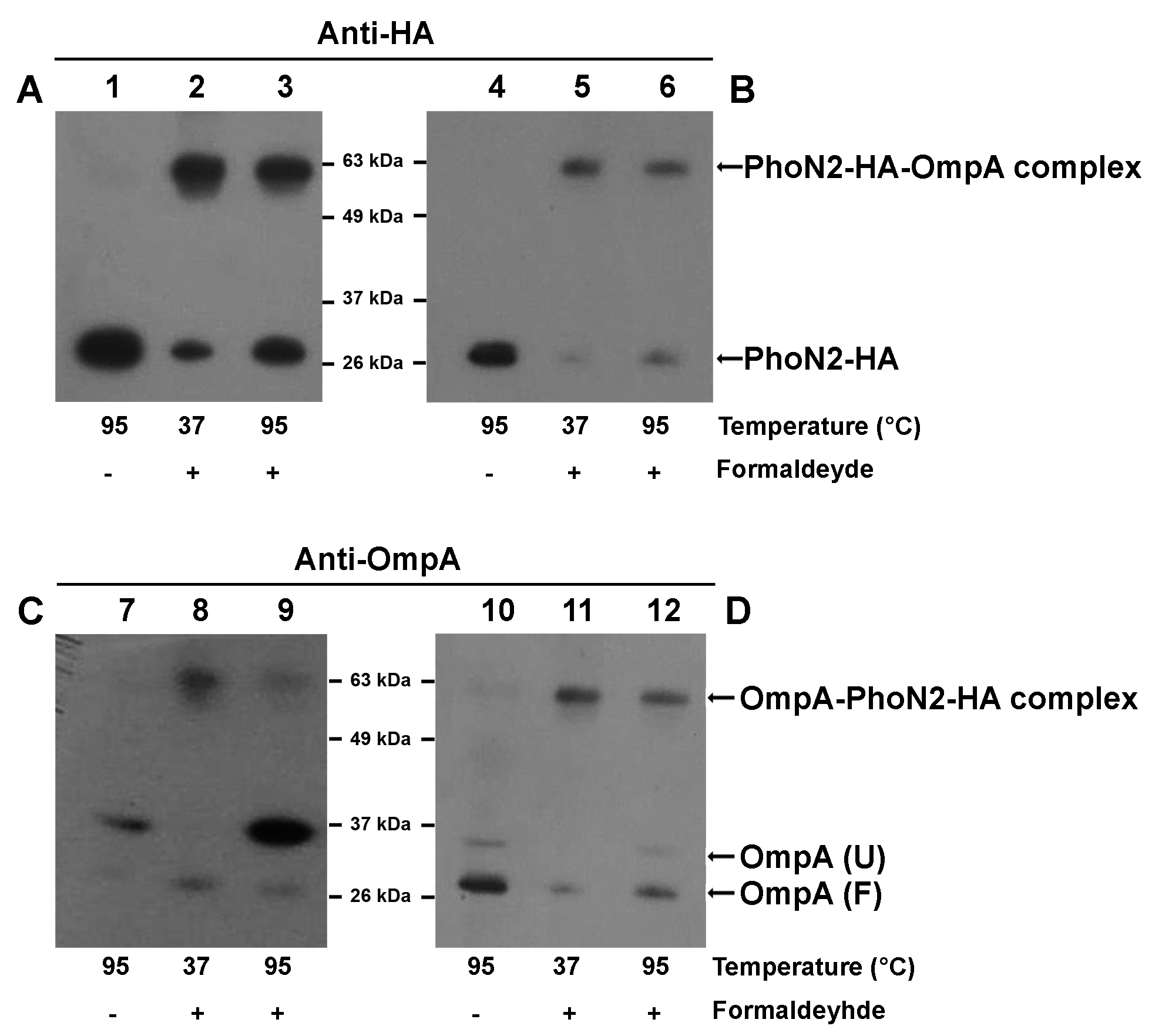

Supplement: Figure S5 — The 183PAPAP187 motif of OmpA is not required for the PhoN2-OmpA interaction. In vivo cross-linking experiments. Cross-linking of the S. flexneri mutant strain HND93, complemented either with plasmids pHND10 and pOmpA (Panels A and C), or with plasmids pHND10 and pAAAOmpA (Panels B and D, Table S1) was achieved by treating bacteria with formaldehyde to a final concentration of 1%, as described in Materials and Methods. Samples were suspended in Laemmli buffer and either heated at 37°C for 10 min to maintain cross-links or at 95°C for 20 min to break cross-links. Equal amounts of proteins were analyzed by Western blot. A protein molecular weight marker (Pierce) was used to determine the molecular weight of proteins. Immunoblotting was carried out using monoclonal anti-HA (Panels A and B) or polyclonal anti-OmpA antibodies (Panels C and D). Expression of phoN2-HA was achieved by growing bacteria in the presence of 0.016% of L-arabinose. OmpA (U), unfolded OmpA; OmpA (F), folded OmpA [48]. The relative position of the PhoN2-HA-OmpA complex is indicated (right). Experiments were repeated at least three times and typical results are shown. (TIF) [file pone.0090230.s005.tif]
