# Supplementary material for: Polar Localization of PhoN2, a Periplasmic Virulence-Associated Factor of Shigella flexneri, Is Required for Proper IcsA Exposition at the Old Bacterial Pole
Source: PLoS One. 2014 Feb 27;9(2):e90230. doi: 10.1371/journal.pone.0090230 (PMC3937361; doi:10.1371/journal.pone.0090230)
Supplement: Table S1 — Bacterial/yeast strains and plasmids used in this work. (DOC) [file pone.0090230.s006.doc]

**Table S1.** Bacterial/yeast strains and plasmids^a^ used in this work.

| **Strain or plasmid** | **Description** | **Source or reference** |
| --- | --- | --- |
| **Strains** |  |  |
| **M90T** | Wild-type *S. flexneri* serotype 5a | Sansonetti *et al*., 1982 |
| **HND115** | M90T Δ*phoN2*; susceptible | Santapaola *et al.*, 2006 |
| **HNDHA10** | M90T *phoN2*::HA; susceptible | This work |
| **HND92** | M90T Δ*ompA*; Km^r^ | Ambrosi et al., 2012 |
| **HND93** | HND115 Δ*ompA*; Km | This work |
| **BS176** | M90T cured of the virulence plasmid | Sansonetti et al., 1986 |
| **SC560** | M90T Δ*icsA*::Ω*;* Sm^r^ | d’Hauteville and Sansonetti, 1992 |
| **DH10b** | *E*. *coli* K-12 F^−^ *mcrA* Δ (*mrr-hsdRMS-mcrBC*) *φ*80d*lacZ*ΔM15 Δ*lacX74 deoR recA1 ara*Δ*139* Δ(*ara leu*)*7697 galU galK λ*^−^ *rpsL endA1 nupG* | Gibco BRL |
| **XL1-Blue** | *E*. *coli* K-12 *endA1* *gyrA*96(nal^R^) *thi-1* *recA1* *relA1* *lac* *glnV44* F'[::Tn*10* *proAB*^+^ *lacI^q^* Δ(*lacZ*)M15] *hsdR17*(r_K_^-^ m_K_^+^) | Stratagene |
| **ME9062** | *E*. *coli* K-12 BW25113 *lacl*^q^ *rrnB*_T14_ Δ*lacZ*_WJ16_ *hsdR514* Δ*araBAD*_AH33_ Δ*rhaBAD*_LD78_ | Baba *et al.*, 2006 |
| **JW0940** | *E*. *coli* K-12 ME9062 Δ*ompA*; | Baba *et al*., 2006 |
| **AH109** | *S.* *cerevisiae* MATa, trp1-901, leu2-3, 112, ura3-52, his3-200, gal4Δ, gal80Δ, LYS2::GAL1_UAS_-GAL1_TATA_-HIS3, GAL2_UAS_-GAL2_TATA_-ADE2, URA3::MEL1_UAS_-MEL1_TATA_-lacZ, MEL1 | Clontech |
| **Plasmids** |  |  |
| **pGEM-T** | cloning vector, Ap^r^ | Promega |
| **pSU315** | Template plasmid carrying an HA epitope and a Km^r^ cassette; Km^r^ | Uzzau *et al*., 2001 |
| **pKD46** | λ Red helper plasmid: *oriR101 repA101*(Ts) *P-araB-gam-bet-exo*; Ap^r^ | Datsenko and Wanner, 2000 |
| **pCP20** | FLP helper plasmid; pSC101 replicon (Ts) *bla* *cat* Flp (λR*p*) *cI*857; Ap^r^ Cm^r^ | Datsenko and Wanner, 2000 |
| **pACYC184** | Low-copy number cloning vector; Tc^r^ Cm^r^ | Fermentas |
| **pBAD28** | Arabinose-inducible P_BAD_ expression vector, Ap^r^ Cm^r^ | Guzman *et al.*, 1995 |
| **pHND10** | pBAD28 carrying *phoN2*::HA; Ap^r^ Cm^r^ | This work |
| **pHND19_R192P_** | pHND10-derivative encoding the PhoN2-HA R192P amino acid substitution; Ap^r^ Cm^r^ | This work |
| **pHND11_Δ79-223_** | pHND10-derivative presenting a 144 nt in-frame deletion (79 to 223) of *phoN2*::HA; Ap^r^ Cm^r^ | This work |
| **pHND23_SPPP_** | pHND10-derivative encoding PhoN2-HA P43S amino acid substitution | This work |
| **pHND14_PSPP_** | pHND10-derivative encoding the PhoN2-HA P44S amino acid substitution; Ap^r^ Cm^r^ | This work |
| **pHND15_PPSP_** | pHND10-derivative encoding the PhoN2-HA P45S amino acid substitution; Ap^r^ Cm^r^ | This work |
| **pHND16_PPPS_** | pHND10-derivative encoding the PhoN2-HA P46S amino acid substitution; Ap^r^ Cm^r^ | This work |
| **pHND21_Y155A_** | pHND10-derivative encoding the PhoN2-HA Y155A amino acid substitution; Ap^r^ Cm^r^ | This work |
| **pOmpA** | pACYC184 carrying *ompA*; Tc^r^ | This work |
| **pAAAompA** | pOmpA encoding the OmpA with P to A substitutions in the ^183^PAPAP^187^ motif; Tc^r^ | This work |
| **pGBKT7** | Cloning vector; TRP1, Km^r^ | Clontech |
| **pGBKT7/*phoN2*** | pGBKT7 carrying the GAL4 DNA-BD fused with the *phoN2* gene; TRP1, Km^r^ | This work |
| **pGADT7-Rec** | *Sma*I linearized cloning vector used to clone the DNA library of M90T; LEU2, Ap^r^ | Clontech |

^a^Ap^r^, ampicillin resistance; Cm^r^, cloramphenicol resistance; Km^r^, kanamycin resistance; Tc^r^, tetracycline resistance.

Sansonetti PJ, Kopecko DJ, Formal SB (1982) Involvement of a plasmid in the invasive ability of *Shigella flexneri*. Infect Immun 35: 852–860.

Santapaola D, Del Chierico F, Petrucca A, Uzzau S, Casalino M et al. (2006) Apyrase, the product of the virulence plasmid-encoded *phoN2* (*apy*) gene of *Shigella flexneri*, is necessary for proper unipolar IcsA localization and for efficient intercellular spread. J Bacteriol 188**:** 1620–1627.

Ambrosi C, Pompili M, Scribano D, Zagaglia C, Ripa S, et al. (2012) Outer membrane protein A (OmpA): a new player in *Shigella flexneri* protrusion formation and inter-cellular spreading. PLoS One.7:e49625

Sansonetti PJ, Ryter A, Clerc P, Maurelli AT, Mounier J (1986) Multiplication of *Shigella flexneri* within HeLa cells: lysis of the phagocytic vacuole and plasmid-mediated contact hemolysis. Infect Immun 51: 461-469.

d’Hauteville H, Sansonetti PJ (1992) Phosphorylation of IcsA by cAMP-dependent protein kinase and its effect on intracellular spread of *Shigella flexneri*. Mol Microbiol 6: 833–841.

Baba T, Ara T, Hasegawa M, Takai Y, Okumura Y, et al. (2006) Construction of *Escherichia coli* K-12 in-frame, single-gene knockout mutants: the Keio collection. Mol Syst Biol 2: 2006.0008.

Uzzau S, Figueroa-Bossi N, Rubino S, Bossi L (2001) Epitome tagging of chromosomal genes in *Salmonella*. Proc Natl Acad Sci USA 98**:** 15264–15269.

Datsenko KA, Wanner BL (2000) One-step inactivation of chromosomal genes in *Escherichia coli* K-12 using PCR products. Proc Natl Acad Sci USA 97: 6640–6645.

Guzman LM, Belin D, Carson MJ, Beckwith J (1995) Tight regulation, modulation, and high-level expression by vectors containing the arabinose P_BAD_ promoter. J Bacteriol 177: 4121–4130.
