# Supplementary material for: Polar Localization of PhoN2, a Periplasmic Virulence-Associated Factor of Shigella flexneri, Is Required for Proper IcsA Exposition at the Old Bacterial Pole
Source: PLoS One. 2014 Feb 27;9(2):e90230. doi: 10.1371/journal.pone.0090230 (PMC3937361; doi:10.1371/journal.pone.0090230)
Supplement: Table S2 — Primers used in this work. (DOCX) [file pone.0090230.s007.docx]

| **Table S2.** Primers used in this work. | | |
| --- | --- | --- |
| **Primer** | **Sequence (5’-3’)** | **Purpose** |
| ^b^PhoN2fw | 5’-TTTGAAGAATTAAATACTCCTACCAATGAACTGACCCC  A*TATCCGTATGATGTTCCTGA-*3’ | Strain HNDHA10 construction (M90T *phoN2*::HA) |
| ^b^PhoN2rv | 5’-TCTATGGGCCCTCCATAGCCTGATACAGGCTGTCCAG  CT*CATATGAATATCCTCCTTAG-*3’ |  |
| ^c^PhoN2HAfw | 5’-GGGGGGGTACCATGAAAACCAAAAACTTTCTTCTTT*-*3’- | Plasmid pHND10 construction |
| ^c^PhoN2HArv | 5’-CCCCCAAGCTTACTAGAGGCTAGCATAATAGGAACATC*-*3’ |  |
| ^d^DS4fw | 5’-CCCCCCCTCGAGGCTAGCGAAGATGCATACTAC*-*3’ | Plasmid pHND11_Δ79-223_ construction |
| ^d^LPrv | 5’-GGGGGGCTCGAGTGCCTTCAGAGCATTTGCTG*-*3’ |  |
| ^d^R192Pfw | 5’-TGAGTTTGGAGAAAGTCCGGTCATCTGCGGTGCGC*-*3’ | Plasmid pHND19_R129P_ construction |
| ^d^R192Prv | 5’-GCGCACCGCAGATGACCGGACTTTCTCCAAACTCA*-*3’ |  |
| ^d^SPPPfw | 5’-CAGTTTGTCAATACTTTCGCCGCCTCCGGC*-*3’ | Plasmid pHND23_SPPP_ construction |
| ^d^SPPPrv | 5’-CCGGAGGCGGCGAAAGTATTGACAAACTG*-*3’ |  |
| ^d^PSPPfw | 5’-CAGTTTGTCAATACTTCCGTCGCCTCCGGCAGAGGATTCA  GTAG*-*3’ | Plasmid pHND14_PSPP_ construction |
| ^d^PSPPrv | 5’-CTACTGAATCCTCTGCCGGAGGCGACGGAAGTATTGACAAA  CTG*-*3’ |  |
| ^d^PPSPfw | 5’-CCAGACAGTTTGTCAATACTTCCGCCGTCTCCGGCAGAGGA  TTCAGTAG*-*3’ | Plasmid pHND15_PPSP_ construction |
| ^d^PPSPrv | 5’-CTACTGAATCCTCTGCCGGAGACGGCGGAAGTATTGACAAA  CTGTCTGG*-*3’ |  |
| ^d^PPPSfw | 5’-CAGTTTGTCAATACTTCCGCCGCCTTCGGCAGAGGATTCAG  TAG*-*3’ | Plasmid pHND116_PPPS_ construction |
| ^d^PPPSrv | 5’-CTACTGAATCCTCTGCCGAAGGCGGCGGAAGTATTGACAAA  CTG*-*3’ |  |
| ^d^Y155Afw | 5’-GGCTATCACTGGCTCTGCTCCCTCTGGTCATGC*-*3’ | Plasmid pHND21_Y155A_ construction |
| ^d^Y155Arv | 5’-GCATGACCAGAGGGAGCAGAGCCAGTGATAGCC*-*3’ |  |
| ^e^ompA2FW | 5’-CGCGGGATCCAGGCTTGTCTGAAGCGGTTT*-*3’ | *ompA* cloning into pACYC184 (plasmid pOmpA) |
| ^e^ompA2RV | 5’-GCGGAAGCTTGGCATTGCTGGGTAAGGAAT*-*3’   \|  \| \| --- \| |  |
| ^d^AAAOmpAfw | 5’-GCTCCGGTAGTTGCTGCAGCTGCGGCAGCGGAAGTACAGACC  AAG*-*3’ | Plasmid pAAAOmpA construction |
| ^d^AAAOmpArv | 5’-CGAGGCCATCAACGACGTCGACGCCGTCGCCTTCATGTCTG  GTTC*-*3’ |  |
| ^f^HTYPHfw | 5’-CCCCCCATGGCCATGAAAACCAAAAACTTTCTTCTTT*-*3’ | Plasmid pGBKT7/*phoN2* construction |
| ^f^HTYPHrv | 5’-GGGGGGATCCTTAAGGGGTCAGTTCATTGGTAGG*-*3’ |  |
| ^g^*nusA*Fw | 5’-TGAAGCCGCACGTTATGAAG*-*3’ | Gene *nusA* amplification for Real-Time PCR (Tran *et al*., 2011) |
| ^g^*nusA*Rv | 5’-TCAACGTAATCGCCCAGGTT*-*3’ |  |
| ^g^QPCRapyF | 5’-GCGGAAATACTTCGACGTGG | Gene *phoN2* amplification for Real-Time PCR |
| ^g^QPCRapyR | 5’-GCAGCTGACCCGACTTTCTCC*-*3’ |  |

^a^Restriction sites are underlined.

^b^Primers used to generate the *phoN2*::HA fusion within the pINV virulence plasmid of *S. flexneri* M90T. Fw primers annealed to the 5’ HA coding sequence, while Rv primers annealed to the 3’ of the Km cassette on plasmid pSU315 (in italic). Primers encompass about 40 nt of the 3’ *phoN2* coding sequence*.*

^c^Primers used to clone the *phoN2*::HA fusion into the expression vector pBAD28.

^d^Primers used to generate deletions and amino acid substitutions.

^e^Primers used to clone the *ompA* gene into plasmid pACYC184.

^f^Primers used to clone the *phoN2* gene into vector pGBKT7*.*

^g^Primers used for Real-Time PCR experiments.

Tran CN, Giangrossi M, Prosseda G, Brandi A, Di Martino ML et al. (2011). A multifactor regulatory circuit involving H-NS, VirF and an antisense RNA modulates transcription of the virulence gene *icsA* of *Shigella flexneri*. Nucleic Acids Res 39: 8122-8134.
